# Prognostic and predictive value of circulating tumor DNA during neoadjuvant chemotherapy for triple negative breast cancer

**DOI:** 10.1038/s41598-020-71236-y

**Published:** 2020-09-07

**Authors:** Luca Cavallone, Adriana Aguilar-Mahecha, Josiane Lafleur, Susie Brousse, Mohammed Aldamry, Talia Roseshter, Cathy Lan, Najmeh Alirezaie, Eric Bareke, Jacek Majewski, Cristiano Ferrario, Saima Hassan, Federico Discepola, Carole Seguin, Catalin Mihalcioiu, Elizabeth A. Marcus, André Robidoux, Josée-Anne Roy, Manuela Pelmus, Mark Basik

**Affiliations:** 1 Lady Davis Institute, Jewish General Hospital, Montreal, QC H3T 1E2 Canada; 2 Department of Human Genetics, McGill University, Montreal, QC Canada; 3 Department of Oncology, Jewish General Hospital, Montreal, QC Canada; 4 Division of Surgical Oncology, Department of Surgery, Centre Hospitalier de L’Université de Montréal (CHUM), Montreal, QC Canada; 5 Department of Radiology, Jewish General Hospital, Montreal, QC Canada; 6 Department of Oncology, McGill University, Montreal, QC Canada; 7 Cook County Hospital, Chicago, IL USA; 8 Hôpital du Sacré-Cœur de Montréal, Montreal, QC Canada; 9 Department of Pathology, Jewish General Hospital, Montreal, QC Canada

**Keywords:** Breast cancer, Tumour biomarkers

## Abstract

Response to neoadjuvant chemotherapy (NAC) in triple negative breast cancer (TNBC) is highly prognostic and determines whether adjuvant chemotherapy is needed if residual tumor is found at surgery. To evaluate the predictive and prognostic values of circulating tumor DNA (ctDNA) in this setting, we analyzed tumor and serial bloods from 26 TNBC patients collected prior, during, and after NAC. Individual digital droplet PCR assays were developed for 121 variants (average 5/patient) identified from tumor sequencing, enabling ctDNA detection in 96% of patients at baseline. Mutant allele frequency at baseline was associated with clinical characteristics. Levels drastically fell after one cycle of NAC, especially in patients whose tumors would go on to have a pathological complete response (pCR), but then rose significantly before surgery in patients with significant residual tumor at surgery (*p* = 0.0001). The detection of ctDNA early during treatment and also late at the end of NAC before surgery was strongly predictive of residual tumor at surgery, but its absence was less predictive of pCR, especially when only TP53 variants are considered. ctDNA detection at the end of neoadjuvant chemotherapy indicated significantly worse relapse-free survival (HR = 0.29 (95% CI 0.08–0.98), *p* = 0.046), and overall survival (HR = 0.27 95% CI 0.075–0.96), *p* = 0.043). Hence, individualized multi-variant ctDNA testing during and after NAC prior to surgery has prognostic and predictive value in early TNBC patients.

## Introduction

Triple negative breast cancer (TNBC) is the most aggressive form of breast cancer and, in the absence of expression of hormone and HER2 growth factor receptors, it is treated with chemotherapy either before surgery (neoadjuvant chemotherapy—NAC), or after (adjuvant chemotherapy). The benefits of NAC include improving chances of breast conservation, limiting the extent of lymph node surgery, as well as providing an excellent prognostic indicator according to the response to NAC with patients responding to NAC having a lower risk of relapse^1,2^. In 2017, the publication of the CREATE-X clinical trial^3^ has resulted in a change in clinical practice, such that patients with TNBCs that have incomplete response to NAC are candidates for further chemotherapy (Capecitabine) for 6 months. Indeed, the addition of Capecitabine results in an improvement in disease-free survival from 56 to 70% at 5 years in TNBC patients. However, these results suggest that over 50% of these patients do not require Capecitabine. So, a need exists to better identify which patients are at highest risk of harboring occult micro-metastases post NAC and could benefit from adjuvant chemotherapy, or conversely those that have an inherently good prognosis and could avoid it. Moreover, the success of neoadjuvant chemotherapy has questioned the need for surgery in cases of complete response^4^, and biomarkers that can predict pathological complete response (pCR) are needed.

One of the most exciting novel biomarkers in cancer diagnostics is the detection of circulating cell free tumor DNA (ctDNA). The identification of somatic DNA variants by tumor sequencing has allowed the highly specific detection of tumor DNA containing the same variants in plasma. This has opened a whole new field of diagnostics where “liquid biopsies” are seen as a less invasive and a less expensive method to monitor disease progression and guide treatment^5,6^. Recent work has shown that ctDNA is detectable in the majority of early common cancers such as breast cancer^7–9^. The presence of ctDNA also has a marked prognostic value when detected after surgical resection^10^ since its presence and quantity post-surgery or treatment reflects the persistence of micro-metastatic residual disease that is otherwise clinically undetectable. Others have shown that the detection of ctDNA is prognostic and also predictive of response to targeted therapies^11–13^. Therefore, ctDNA could potentially be an excellent marker of residual disease in patients with TNBCs, and could be used to guide therapeutic decisions after NAC. However, many of the present ctDNA approaches rely on targeted mutation analysis of a few frequently mutated genes with mutation hotspots, an approach hardly applicable to TNBCs since they have few recurrent mutations except for those in the TP53 gene. Indeed, the remarkable tumor DNA heterogeneity of TNBCs makes it difficult to use a one size fits all approach, and requires the development of specific biomarkers individualized to each tumor.

We recently completed the Q-CROC-03 clinical trial, in which patients with TNBC undergoing NAC were consented for biopsies pre-chemotherapy and post-chemotherapy as well as serial bloods before, during and after NAC^14^ to determine molecular factors of response or resistance to standard NAC both in tumor tissue and plasma. We also recently reported on optimized protocols for cfDNA extraction and digital droplet polymerase chain reaction (ddPCR) analysis^15^. We applied these protocols to the analysis of serial plasma from 26 patients enrolled in Q-CROC-03, by developing personalized ddPCR assays based on whole exome sequencing (WES) of each tumor. We herein present our findings, which confirm the power of ctDNA as a strong potential prognostic and predictive biomarker in the early stages of this aggressive disease.

## Results

### Q-CROC-03 patients and bloods

**Figure 1:**
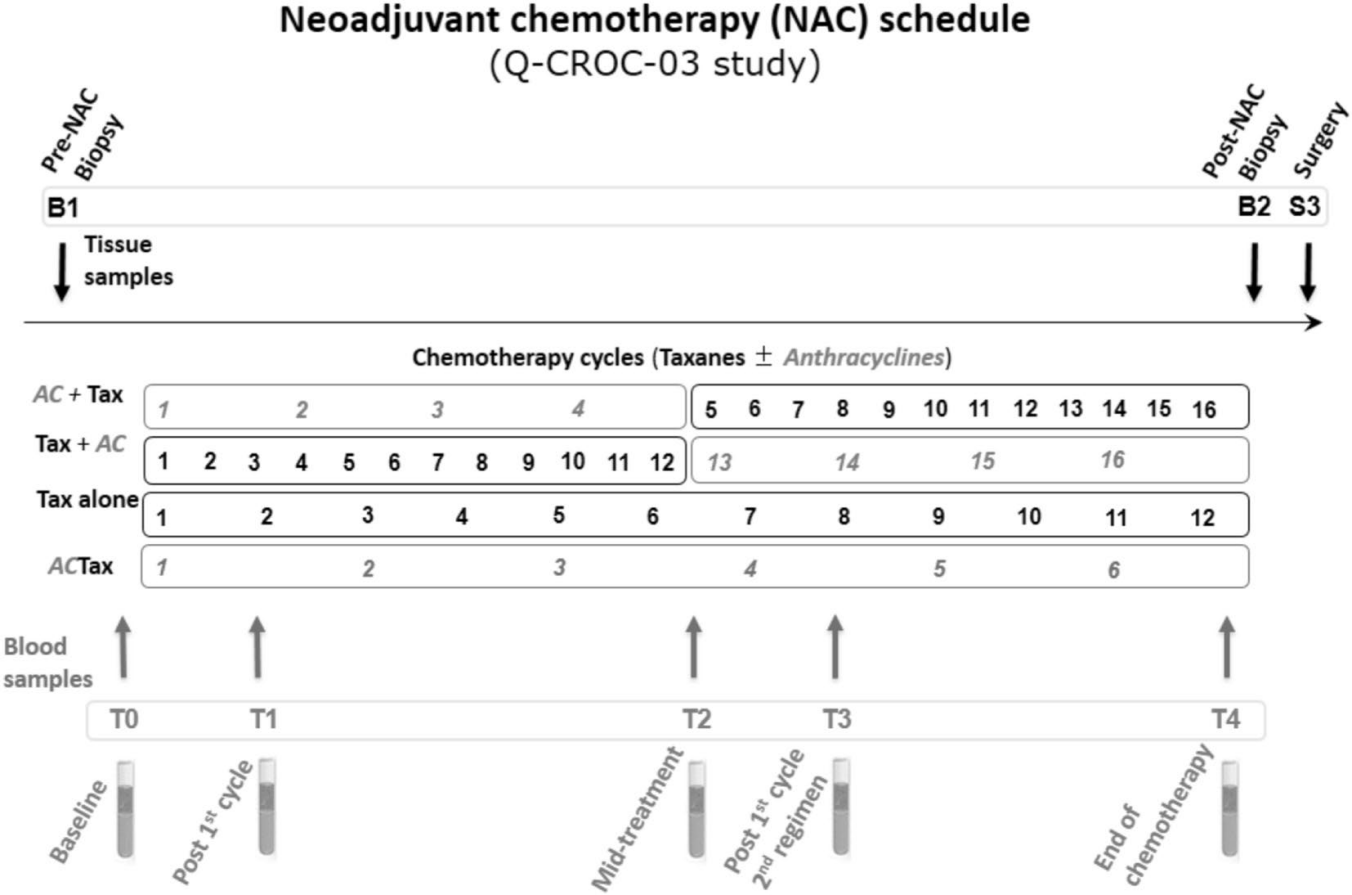
Neoadjuvant chemotherapy (NAC) and biobanking schedule of the Q-CROC-03 study. An illustration of the schedule of various chemotherapy regimens with the biobanking collection schedule superimposed. Tissue sample collection occurred before the start of NAC at the time of diagnosis (B1), and then either after NAC (B2) and/or at surgery (S3) in the case of non-pCR patients. Up to 5 blood samples were collected: 1 before the start of NAC at baseline (T0), 1 after the 1st cycle of the 1st drug regimen (T1), 1 at mid-treatment or at the switch between the 2 drugs regimen (T2), 1 after the 1st cycle of the 2nd drug regimen (T3), 1 at the end of NAC (T4) before surgery). The different NAC drug regimens were AC + Tax [4 cycles of Doxorubicin (or Epirubicin) with Cyclophosphamide followed by 12 cycles of weekly Paclitaxel, or 3 cycles of 5-Fluorouracil/Epirubicin/Cyclophosphamide followed by 12 cycles of weekly Paclitaxel (or 3 cycles of Docetaxel)]; Tax + AC (12 cycles of weekly Paclitaxel followed by 4 cycles of Doxorubicin (or Epirubicin) with Cyclophosphamide, or 3 cycles of 5-Fluorouracil/Epirubicin/Cyclophosphamide); Tax only (12 cycles of weekly Paclitaxel); or ACTax (3 or 4 cycles of Doxorubicin with Cyclophosphamide + Docetaxel, or 4 cycles of Carboplatin/Paclitaxel).

The Q-CROC-03 clinical trial enrolled patients with TNBC undergoing standard anthracycline/taxane or taxane-alone neoadjuvant chemotherapy (NAC). Patients consented to pre and post-chemotherapy biopsies and surgical tissue collection [for whole exome sequencing (WES)] as well as to serial blood sampling at 5 time points: prior to NAC, after 1 cycle of NAC, at mid-treatment (between the anthracycline and taxane components), after 1 cycle of the 2nd chemo regimen and at the end of NAC before surgery (Fig. 1). 60 patients were enrolled in this study but only 26 were eligible for this study with high quality tumor DNA sufficient for WES, as well as at least 2 serial blood samples collected according to established SOPs (Supplementary Fig. S1 and Supplementary Table S1). The present report presents data on these 26 patients, with their clinical characteristics shown in Supplementary Table S2 and the time points at which blood samples were collected shown in Supplementary Table S1 and Fig. 1. Eight of these 26 patients achieved pathological complete response (pCR) or Residual Cancer Burden 0 (RCB 0), 3 achieved near-complete response (RCB 1) as per the Symmans et al.^2^ criteria, while 14 had significant residual tumor present at surgery (RCB 2–3), and one patient who developed metastasis and did not have surgery was considered as RCB 3 (Neo30).

### Variant selection and detection

We selected the genes containing variants based on 3 criteria: (1) genes with the highest MAFs in the tumors, (2) TP53 gene variants and (3) genes in which the MAFs “changed” from pre to post-NAC (either from 0 pre-NAC to a high value post-NAC or, the opposite, from a high value pre-NAC to 0 post-NAC) in the tumor (Supplementary Table S3). ddPCR assays were developed for a total of 121 variants (average of 5/tumor, range 1–12/tumor), and each one was validated by performing ddPCR on tumor DNA. The correlation between ddPCR and WES variant allele frequencies in tumors was excellent (r^2^ = 0.73, *p* < 0.0001) (Supplementary Fig. S2). The TP53 gene was the only gene frequently mutated in this cohort (69% of patients), while RB1 and ROBO2 were both mutated twice with different variants. ddPCR assays were developed for variants in the TP53 gene in all 18 patients with tumors containing a TP53 gene mutation, and only 2 variants were shared in 2 patients each (Supplementary Table S3), making these the only recurrent variants in this cohort. Once ddPCR assays were validated in corresponding tumor tissue, ddPCR assays were performed on all blood samples from that patient for a total of 149 blood samples analyzed in this study. The median follow-up of the cohort is 63 months post-diagnosis and 55 months post-surgery.

### Thresholds of detection for ctDNA

We defined a “normal plasma” threshold value for each variant individually. To this end, we created 3 pools of plasma from 10 normal cancer-free individuals, and each pool was used to test the ddPCR assays of 110 variants that were detectable (MAF values above 0) in at least one of the Q-CROC-03 blood samples. We observed a broad range of MAF in normal plasma, from 0 (27% of variants) to 0.32%, with a median of 0.02% (Supplementary Table S4). The levels of MAF detected may be due to the pre-amplification step in our protocol^15^. These targeted pre-amplifications, which involved the generation of specific PCR primers for each DNA fragment containing the selected variant, were performed for each variant in a multiplex PCR, enabling the testing of multiple variants from the same DNA sample with limited usage of the precious DNA per variant tested. As we reported, such “targeted pre-amplification” shows consistently similar MAFs compared to non-pre-amplified samples, even in samples with MAFs as low as 0.05%^15^. In order to ensure the reliability of the calling of “detection”, we established stringent criteria using a pool of normal samples: a positive ctDNA value had to be 2 standard deviations above the mean of values in 3 normal pools. We observed that the values in the 3 pools were quite consistent, with a small average standard deviation of 0.017%, suggesting that these “false positives” were likely intrinsic to each specific ddPCR assay. Focusing only on TP53 gene variants, the range in normal plasma samples was 0 to 0.29%, with a median of 0.04%. The threshold for a called or “detected” variant was set as the mean of the 3 pool values + 2 standard deviations above it. Using these criteria, we detected at least one variant in at least one time point during NAC in 96% of patients in our cohort (25/26 patients). In total, 95 of 121 total variants (80%) were called detected (i.e. above threshold) in the plasma of these 25 patients (Supplementary Table S5). In one patient (Neo58) none of the variants were detected above threshold in any of the serial plasma samples. This patient had a stage 2 tumor (T2N1M0) and achieved a complete response (pCR) after NAC.

### Baseline ctDNA

**Figure 2:**
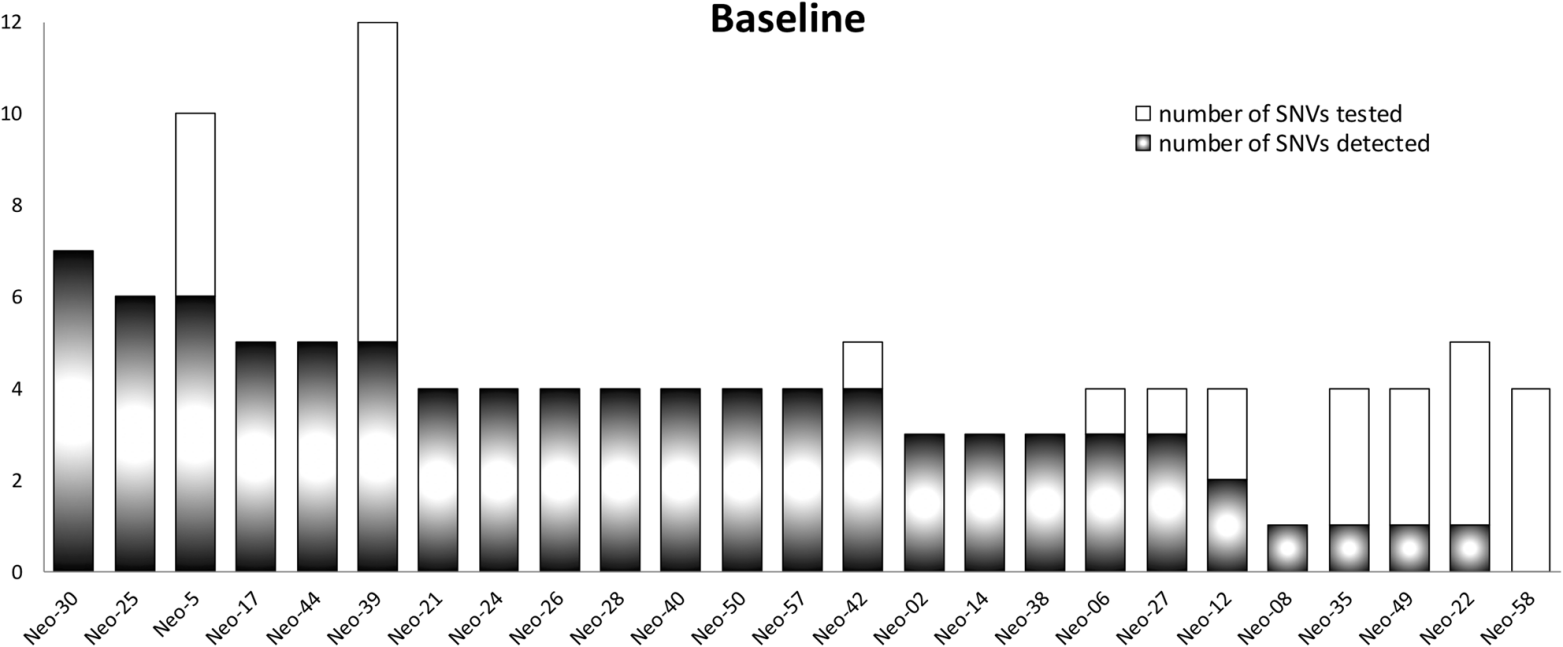
Detection of Single Nucleotide Variants (SNVs) at baseline (T0). For each patient, the total height of the bar represents total number of variants tested. Shaded (black) area represents the number of detected variants at T0.

One patient (Neo07) did not provide blood samples prior to the start of NAC. We detected ctDNA (at least one variant above threshold level) in the baseline pre-chemotherapy (T0) blood sample in 24 of 25 patients (96%) (Fig. 2), and 19 (76%) had at least 3 variants detected, while 4 (16%) had only one (Fig. 2). The number of variants detected per patient ranged from 0 to 7, with a median of 4 variants. In total, 87 variants out of 117 (74%) tested at baseline were detected and, assigning non-detectable variants the value of 0, the average MAF in the baseline (pre-NAC) plasma from 25 patients was 2.86%, with a median of 0.41% for all variants tested and 0.81% for the 87 variants detected (> threshold value) at baseline (Table 1). The mean MAF at baseline was significantly correlated with clinical factors such as tumor size (cT), stage, grade, nodal status at diagnosis (cN) or surgery (pN), and RCB score, but not with patient age (Table 2). For instance, the mean baseline MAF of patients with T3–T4 tumors was 11 times higher than for those with smaller tumors (*p* = 0.01), and the MAF of patients with clinically positive axillary nodes (cN1-3) was fourfold higher than in those with cN0 (*p* = 0.02). Similarly, the mean baseline MAF of patients with RCB > 0 was fivefold higher than in those with a complete tumor response to NAC (RCB 0 or pCR) (*p* = 0.0065). We did not observe a relationship between MAF in tissue samples and response to therapy (Supplementary Figure S3). These results suggest that the anatomical extent of the primary tumor (i.e. primary tumor burden) is highly correlated with the amount of baseline ctDNA detectable in the plasma of TNBC patients when an average of 5 variants per patient are used to detect ctDNA.

**Table 1:** Mutant Allele Frequencies dispersion values over different time points.

Variants considered	Measures of MAF dispersion	T0	T1	T2	T3	T4
All variants tested	Average	2.86	0.39	0.11	0.13	0.42
	Standard deviation	8.26	0.97	0.45	0.41	1.12
	Median	0.41	0	0	0	0
	Min–Max	0–52	0–5.4	0–3.8	0–2.6	0–5.7
87 detected at baseline	Average	3.84	0.53	0.15	0.17	0.49
	Standard deviation	9.39	1.11	0.53	0.48	1.26
	Median	0.81	0.05	0	0	0
	Min–Max	0.06–52	0–5.4	0–3.8	0–2.6	0–5.7
TP 53 variants	Average	4.66	0.47	0.30	0.20	0.60
	Standard deviation	11.28	1.29	0.98	0.45	1.37
	Median	0.63	0.07	0	0	0
	Min–Max	0–45.7	0–5.4	0–3.8	0–1.7	0–5.1

**Table 2:** Association of Average Mutant Allele Frequencies at baseline (T0) with patient and tumor characteristics.

Clinical characteristics			Patients, n (%)	Average MAF at baseline for all variants (%)	*p* value
**Patients**	*Age, years*	≤ 50	15 (58)	1.89	0.2010
		> 50	11 (42)	3.90	
	*BRCA 1/2 mutated*^a^	Positive	4 (57)	1.76	0.0245
		Negative	3 (43)	0.34	
**Pre-NAC** (Neoadjuvant chemotherapy) **tumor**	*cT*	1–2	21 (81)	1.05	0.0127
		3–4	5 (19)	11.65	
	*cN*	0	13 (50)	1.10	0.0216
		1–3	13 (50)	4.78	
	*Stage*	I	*1 (4)*	*0.01*	*Only 1 value*
		II	19 (73)	1.15	0.0167
		III	4 (22)	9.73	
	*Grade*	1	*0 (0)*		*No value*
		2	5 (19)	0.42	0.0019
		3	21 (81)	3.30	
	*Histological type*^b^	Ductal	25 (89)	3.00	0.0842
		Lobular	3 (11)	13.26	
**Treatment**	*NAC drugs regimen*	Only taxane	4 (15)	0.51	0.2392
		AT	17 (65)	3.94	
		TA	3 (12)	0.80	
		Other	2 (8)	4.39	
**Post-NAC tumor**^c^	*pN*	Negative	17 (68)	1.15	0.0207
		Positive	8 (32)	6.24	
	*Residual tumor size (cm)*	0	9 (36)	0.64	0.0626
		≤ 2	4 (16)	1.39	
		> 2	12 (48)	4.69	
	*RCB*	0	8 (31)	0.69	0.0065
		≥ 1	18 (69)	3.57	
		0–1	11 (42)	0.96	0.0146
		2–3	15 (58)	3.90	

Average MAF of all 117 gene variants according to different patient characteristics (age, BRCA1/2 status), pre-neoadjuvant chemotherapy (pre-NAC) tumor characteristics (cT, cN, stage, grade, and histology), treatment (NAC), or post-NAC tumor characteristics (pN, residual tumor size, RCB score). Stage (cT, cN and stage) is according to the AJCC 8th edition. NAC drug regimens were classified as only taxanes, anthracycline before (AT) or after (TA) taxanes, or other regimens. Average MAF is compared with a two-tailed Student’s *t* test when there were 2 independent groups and with an analysis of variance (ANOVA) test when there were more than 2. Italic is added when there are not enough values to do the calculation and there are no *p* values < 0.05.

^a^BRCA1/2 status: unknown data for 19, so we have n = 7.

^b^2 patients had lobular and ductal breast cancer, so we have n = 28.

^c^One patient had no surgery due to the development of metastatic disease, so we have n = 25 at the surgery for pN and residual tumor size. This patient was assigned an RCB score = 3.

### Early response ctDNA data

**Figure 3:**
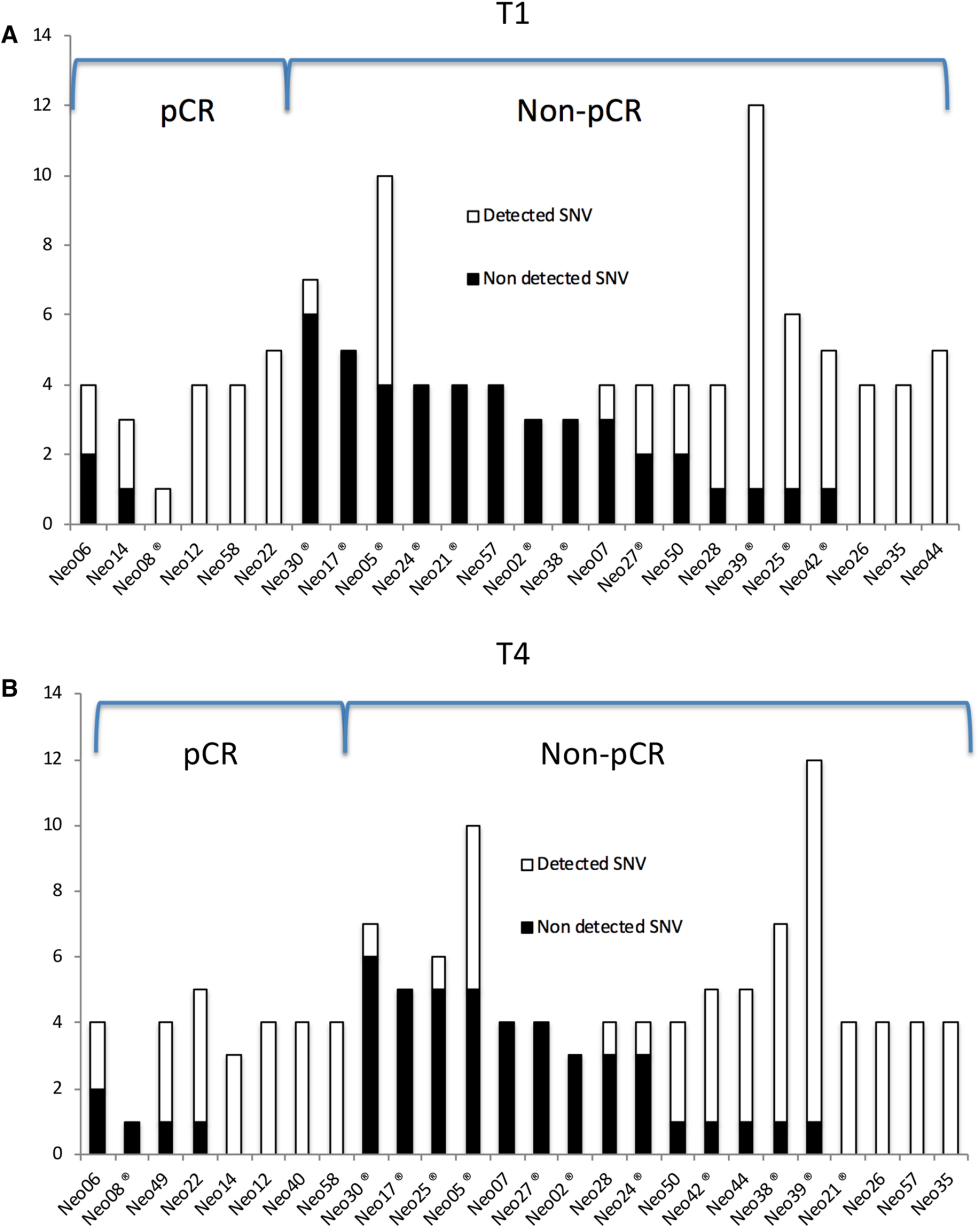
Detection of SNVs after 1st cycle of NAC (T1) and at the end of NAC (T4). Total height of bars represents total number of tested variants for each patient. Shaded area of each bar represents the number of detected variants at T1 (**A**) and at T4 (**B**) in pCR and non-pCR (RCB > 0) patients. X-axis labels refer to the patient identifier (ID), ® refers to patients with breast cancer relapse.

We measured the ctDNA levels of 98–121 variants at 1 to 4 different time points during NAC. All but two patients (Neo40 and Neo49) provided a first “on-treatment” blood drawn just prior to the second cycle of chemotherapy (T1). At this time point, ctDNA was detectable (at least one variant) in 17 of 24 (71%) patients (Fig. 3A), and associated with the presence of residual disease at the time of surgery: 33% (2 of 6) patients with pCR had detectable ctDNA compared to 83% of patients with residual tumor (15 of 18) (Fisher exact *t* test *p* = 0.038) (Fig. 3A). When only patients with variants detected at baseline were included in the analysis, ctDNA was detected at T1 in 40% (2/5) of pCR patients versus 82% (14/17) of non-pCR patients (*p* = 0.10). The positive predictive value (PPV) of ctDNA detection at T1 for the presence of residual disease post NAC was 88% while the negative predictive value (NPV) was 50% when only detected variants at baseline were considered (Table 3A).

**Table 3:** Positive and negative predictive values of ctDNA detection at T1 and T4 for pCR (A) and relapse (B).

	pCR (n)	Non-pCR (n)	PPV^a^	NPV^b^
**A**				
*T1 (87 variants)*				
ctDNA+	2	14	88	
ctDNA−	3	3		50
*T4 (87 variants)*				
ctDNA+	2	13	87	
ctDNA−	5	4		56
*T1 (TP53 variants)*				
ctDNA+	2	7	78	
ctDNA−	1	7		13
*T4 (TP53 variants)*				
ctDNA+	1	7	88	
ctDNA−	3	7		30

	Relapse (n)	Total (n)	PPV^a^	NPV^b^
**B**				
*T1 (87 variants)*				
ctDNA+	10	15^c^	67	
ctDNA−	1	6		83
pCR	1	5		80
Non-pCR	10	16^c^	63	
*T4 (87 variants)*				
ctDNA+	10	14^c^	71	
ctDNA−	1	9		89
pCR	1	7		86
Non-pCR	10	16^c^	63	

ctDNA+, at least 1 variant detected per patient; ctDNA−, no variant detected for any gene.

^a^Positive predictive value: ability of ctDNA+ to predict non- pCR or relapse.

^b^Negative predictive value: ability of ctDNA− to predict pCR or freedom from relapse.

^c^One patient (Neo-30) was not included in association with relapse since the patient was metastatic.

**Figure 4:**
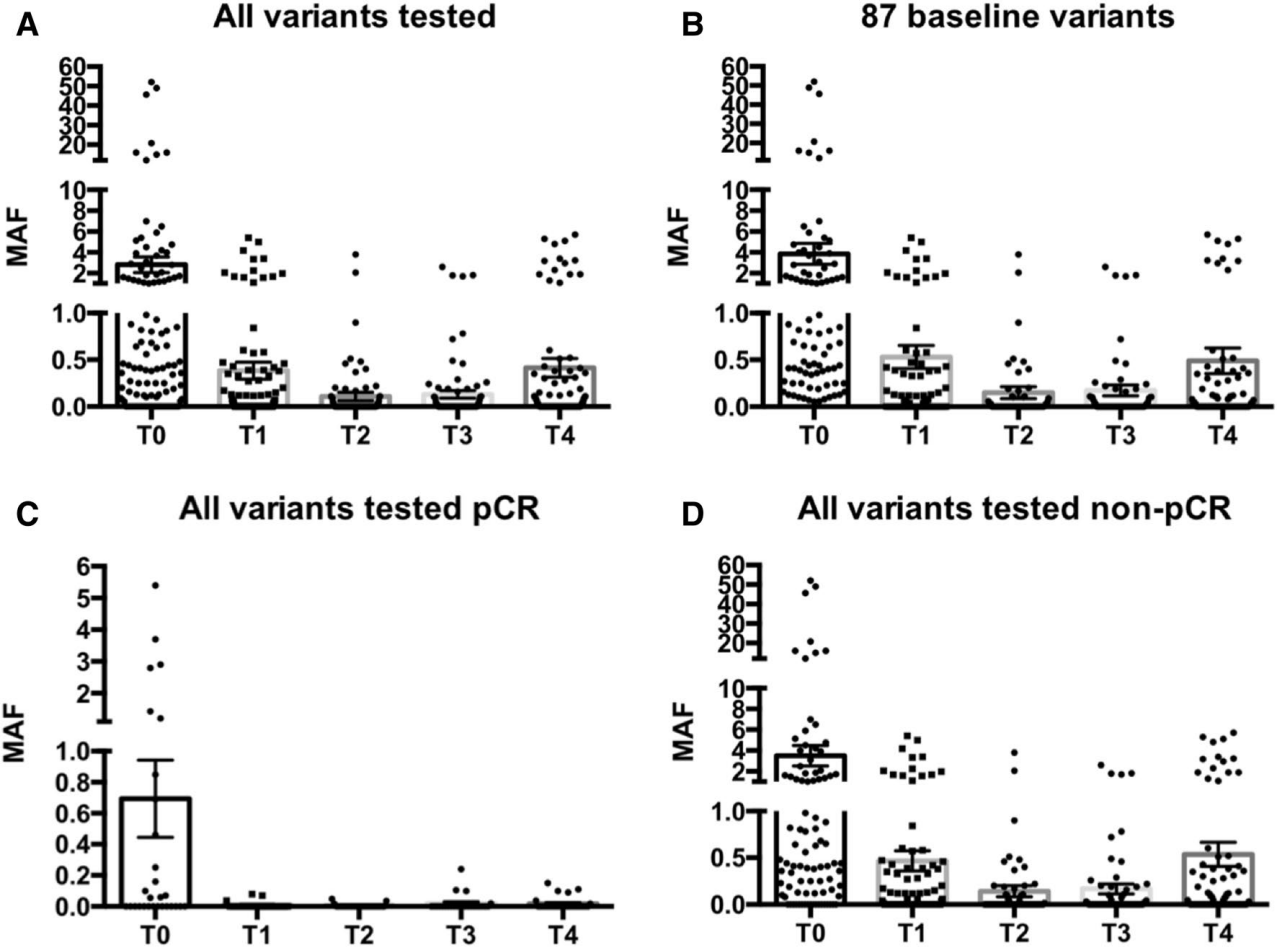
Change in average Mutated Allele Frequencies (MAF) at different time points. Average and SEM of MAFs at each time point for all variants tested (**A**) and for the 87 variants detected at baseline time point T0 (**B**). Average MAFs for all variants tested in patients with pCR (**C**) and non pCR (**D**). Only tested variants are considered at each time point and non-detectable values are assigned a “0” value.

Remarkably, MAF levels dropped dramatically from baseline to T1, falling by 86% to an average MAF of 0.39% for all variants (Fig. 4A, Table 1) and to an average MAF of 0.53% for variants detected at baseline (Fig. 4B, Table 1). This marked fall at T1 was not associated with long-term outcome (*p* > 0.3) as ctDNA fell markedly in all but 2 patients. In one patient (Neo57), the levels of all 4 detected variants rose at this first on-treatment time point, before falling to undetectable levels at later one and she went on to have a pCR. In another patient (Neo24), 2 of the 4 detected variants showed a slight rise at T1, concordant with residual tumor at the time of surgery (RCB 3). It is noteworthy that of 3 patients who did not show any clinical response at mid-cycle (Neo27, Neo30 and Neo50), mean ctDNA levels also fell markedly (mean of 80.4%), suggesting that the early decrease in ctDNA levels is not associated with early primary tumor response (data not shown). Mid-cycle ctDNA levels at T2 were even lower than at T1 for all patients (Fig. 4A,B, Table 1), and 2 of 8 patients (25%) who had pCR and available blood samples at T2 showed detectable ctDNA levels compared to 12 of 15 (80%) for non-pCR patients (*p* = 0.023).

### End of chemotherapy pre-surgery ctDNA

The last blood draw during treatment was collected within a month prior to surgery and at the end of chemotherapy (T4). Eighteen of 26 patients (69%) showed detectable ctDNA for at least one variant (Fig. 3B), and 48 of the 121 variants tested (40%) were detected, including three variants that were not detectable at baseline. Detection of ctDNA at this pre-surgery time point was not significantly associated with poor response to NAC and presence of residual disease at the time of surgery, as it was detected in 4 of the 8 pCR patients (50%) versus 14 of the 18 non-pCR ones (82%) (*p* = 0.20) (Fig. 3B). However, when counting the percentage of detected variants over all the measured ones, patients with pCR had significantly fewer variants detected at this time point. Indeed, only 5 of 29 variants (17%) were detected in the pCR patients, versus 43 of 92 variants (47%) in non-pCR patients (*p* = 0.005).

If we then narrow the analysis to the 24 patients with the 87 variants detected at baseline, 29% of the pCR patients (2/7) had detectable ctDNA at T4 versus 76% of non-pCR patients (13/17) (*p* = 0.061). Including only variants detected at baseline and at T4, the PPV for any variant detected at T4 for the presence of residual disease at the time of surgery was 87% and the NPV of no ctDNA detection for pCR was 56% (Table 3A).

The mean MAF at this final pre-surgery time point is similar to that after the first chemotherapy cycle but higher than mid-cycle values (T2 and T3) (Fig. 4A,B). Interestingly, in pCR patients, the average MAF of all variants tested at T4 remained low (0.017%) and unchanged from T2–T3, while in non-pCR patients, it was about threefold higher (0.55%) than at the T2–T3 time points and more than 30 fold higher than in pCR patients (*p* = 0.0001) (Fig. 4C,D).

### Post-surgery ctDNA

As part of our institutional biobank we had also collected post-operative bloods on 13 Q-CROC-03 patients, from whom 10 had a blood drawn less than 3 months after surgery, 7 with non-pCR, and 3 with pCR. A total of 30 variants were measured in the post-surgery sample of these 10 patients (Supplementary Table S6). In total, we detected 18 variants in the post-surgery blood of 5 of the10 patients and all were detected in non-pCR patients. When we compare to T4 in this same 10 patient cohort, we detect 16 variants in 6 out of 10 patients, all non-pCR patients except for one variant detected in Neo08. Interestingly, the Neo08 patient had a pCR at surgery, but developed a tumor relapse within a year after surgery. The one tested ctDNA variant was only detected at T4 and not post-operatively. Detection of ctDNA prior to and post-surgery was mostly concordant, with all but two variants detected at T4 being detected post-operatively (except for Neo08, and Neo50), and conversely, all but four variants detected post-operatively being detected at T4 (except for Neo25, Neo38, and Neo42). Combining the 2 time points, on a patient basis, these discordances would have changed the ctDNA detection status in 3 patients (Neo-08, Neo-42 and Neo-50), all of which had only 1–3 variants tested at these time points. Overall, the average MAF increased from 2.2% at T4 to 17.8% in the post-operative sample (*p* = 0.023). These limited results suggest that, although the post-operative bloods drawn within 3 months have markedly higher quantities of ctDNA when detected, the rate of pre-operative variant detection was similar to the rate of post-operative one, with discordance becoming significant in those patients with fewer variants tested at these last time points.

### TP53 variant analysis

Since many ctDNA reports are based on pre-selected cancer gene panels containing recurrently mutated genes, and since TP53 was the most frequently mutated gene in our cohort, we performed the above analyses using only the 18 TP53 variants measured in plasma, of which 2 were shared by 2 different patients (p.R81X and p.R150W) (Supplementary Table S7). TP53 variants were detected at baseline in 15 of 17 (88%) patients tested for TP53 and with blood collected at baseline. From the point of view of the entire cohort, this means that 15 of 25 patients (60%) had a positive TP53 ctDNA test at baseline. Unlike the entire group of variants, the baseline MAF of TP53 variants was not associated with clinical characteristics such as tumor size (cT), nodal status (cN and pN), stage, grade or RCB score (Supplementary Table S8). Detection of TP53 variants at T1 was not associated with pCR either, since 7 of the 14 non-pCR patients had detectable TP53 variants (50%), while 2 of 3 of the pCR patients also had detectable ones. At the last time point prior to surgery (T4), ctDNA for TP53 was measured in 18 patients and was detected in 7 of the 14 non-pCR (50%) patients and in 1 of the 4 pCR patients (25%) (*p* = 0.59) (Supplementary Table S7). The PPV of TP53 ctDNA detection at T1 and T4 for the presence of residual disease post NAC (non-pCR) was 78% and 88% respectively while the negative predictive value (NPV) was only 13% and 30% respectively (Table 3A), a result inferior to the ones observed when WES was used to identify private variants for each tumor.

The average MAF of the TP53 ctDNA variants was 4.66% at baseline, 0.47% at the first visit (T1) and 0.60% at the last visit post-chemotherapy (T4), slightly higher than the MAF for the entire set of variants (Table 1).

### Long-term outcome data

**Figure 5:**
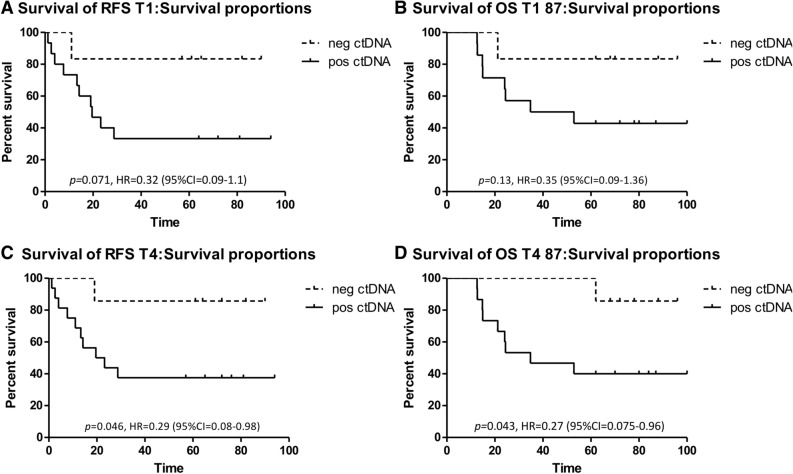
Survival Analysis based on ctDNA detection at T1 and T4 time points. Kaplan–Meier curves of relapse-free survival (RFS) (**A**, **C**) and overall survival (OS) (**B**, **D**) comparing patients for whom we can detect at least 1 SNV at the indicated time point versus the ones for whom we can’t detect any at the same time point. (**A**, **B**) represent RFS and OS at T1, respectively; (**C**, **D**) represent RFS and OS at T4, respectively. In these analyses, we only considered the 80 variants detected at baseline in 23 patients. *p* value is calculated from a log-rank.

During a median follow-up of 63 months post-diagnosis and 55 months post-surgery, 12 patients (46%) developed disease relapse and 10 (38.5%) died of breast cancer (Supplementary Table S2). All but one (Neo08) of the 8 pCR patients are free of disease at last follow-up, while 11 of the 18 non-pCR patients are dead of disease. We removed Neo30 from further outcomes analysis as metastatic disease was detected prior to surgery post-NAC and the patient was not operated. We evaluated the effect of ctDNA detection on relapse-free survival (RFS) and overall survival (OS) using only the 80 variants detected at baseline in the remaining 23 patients, since these are the variants that can be followed throughout the treatment course. At the first chemotherapy cycle (T1), we found that the detection of ctDNA was not significantly associated with either RFS ]*p* = 0.071, HR = 0.32 (95% CI 0.09–1.1)] nor with OS [*p* = 0.13, HR = 0.35 (95% CI 0.09–1.36)] (Fig. 5A,B), although there is a trend towards better survival with no detectable ctDNA at T1. The detection of ctDNA at this time point had a PPV for disease relapse of 67% and the absence of any detectable ctDNA an NPV of 83% (Table 3B). At the end of chemotherapy (T4), the detection of ctDNA was a significant predictor of both RFS and OS (*p* = 0.046, HR = 0.29 (95% CI 0.08–0.98), and *p* = 0.043, HR = 0.27 (95% CI 0.075–0.96) respectively) (Fig. 5C,D). The median time to relapse or to death for patients with detectable ctDNA at T4 was 21 and 35 months respectively and was not reached for patients with no ctDNA detected. PPV of ctDNA detection for disease relapse at this time point was 71%, and NPV of no ctDNA detection for no relapse was 89%. Interestingly, the NPV of pathological assessment of pCR for relapse was 86%, while the PPV of non-pCR for relapse was 63%, both inferior to the predictive values of ctDNA at T4 (Table 3B). Thus, the absence of ctDNA at this pre-operative time point was a marginally better predictor of good outcome than pathological complete response. We also tested the predictive value of RCB for relapse in this small cohort, grouping RCB 0 with RCB 1, as both have excellent prognosis, versus RCB2 and RCB3 tumors. We found the RCB score to have NPV and PPV of 80% and 71% for relapse-free survival and disease relapse, respectively, inferior to the detection of ctDNA at the T4 time point.

## Discussion

Chemotherapy is the mainstay of therapy in triple negative breast cancer, and the response to NAC is one of the strongest prognostic factors in this disease. A high residual cancer burden after NAC identifies a poor prognostic group, for which further treatment is desirable, as shown by the recent publication of the CREATE-X trial in which the administration of 6 months of Capecitabine in TNBC patients who had not had a pCR resulted in an improvement in overall and disease-free survival^3^. The presence of circulating tumor DNA reflects tumor burden in the metastatic setting and increases in ctDNA levels appear to anticipate disease progression^16,17^. Moreover, Garcia-Murillas et al.^10^ recently showed that the persistence of ctDNA after surgery and NAC is a portent of poor prognosis in breast cancer. This group, like ours, first sequenced the tumor, and then developed personalized digital PCR assays for 1–2 variants per patient. The great majority of patients had variants detected in 2 genes: TP53 and PIK3CA. To better understand the sensitivity of ctDNA in TNBCs, we used whole exome sequencing data in 26 TNBCs to generate an average of 5 personalized assays per patient, which we tested against a panel of 30 control plasma samples obtained from cancer-free patients in order to ascertain their specificity. We found a range of MAF from undetectable to 0.32%, with a median of 0.024%, in the normal plasma pools, which are likely due to the pre-amplification step used in our published protocol^15^.

Correcting for the values obtained in our normal plasma pools, we were able to obtain 96% sensitivity, detecting almost all of these early TNBCs. Four patients had only 1 of 4 variants detected, and the 2 TP53 variants in these 4 patients were not among the detectable variants. Indeed, if we had used only TP53 variants, we would have obtained a sensitivity of 60%, somewhat lower than the 75% sensitivity obtained by Riva et al.^9^ in TNBC patients using TP53 variants alone. Our results suggest that in TNBCs, maximizing the sensitivity of the assay requires several assays per patient, and not only selecting recurrently mutated genes. We assessed the sensitivity of fewer variants, for instance choosing only 2 variants, the ones with the highest MAFs in the tumor tissue. Using this approach, we lost sensitivity (e.g. at the T4 time point, 4 of the 26 ctDNA values changed from detectable to non-detectable). Moreover, unlike Garcia-Murillas et al.^10^, the relative proportion of ctDNA (as Mean Allele Frequency) in the plasma at baseline prior to chemotherapy was strongly associated with tumor features such as tumor size, grade, stage, nodal status, and response to NAC, but not when only the TP53 variants were used. Our results are thus more similar to those of Rothé et al.^18^ in which the detection of PIK3CA and/or TP53 variants in the plasma at baseline in HER2+ breast cancers was associated with poorer neoadjuvant treatment response.

We observed that the MAF of variants fell precipitously after the first chemotherapy cycle (T1), independently of tumor response and eventual pathological response. Indeed, the early decrease in ctDNA levels appears to be mostly the result of the effect of chemotherapy on the primary tumor’s ability to generate ctDNA, and not on initial tumor response. Butler et al.^19^, who showed similar results, suggest that chemotherapy may selectively kill actively dividing cells, which are the majority secretors of ctDNA. Nevertheless, those patients whose tumors eventually showed complete pathological response were less likely to have detectable ctDNA at this early stage of neoadjuvant treatment (33% vs 83%). Strikingly, the detectability of ctDNA at this early 3-week time point carried prognostic value, with an ability to predict relapse free recurrence of 86%. These results are similar to those obtained by Riva et al.^9^ in which the presence of TP53 mutations alone in ctDNA was highly prognostic at a similar time point. At the post-chemotherapy time point (T4), the MAF, albeit still very low, began to rise marginally in patients with residual disease, while remaining low or undetectable in those with eventual pCR (> 50 fold lower than non-pCR patients). These results are somewhat different from those of Riva et al.^9^ in which the detection of ctDNA was not associated with pathological response to NAC. However, the absence of ctDNA detected prior to surgery in our series had a NPV for pCR of 56%, a result that, as a stand alone test, it could not be useful to select patients to avoid surgery in upcoming no-surgery trials^4^. Our results are similar to those of McDonald et al., who developed a multiplex targeted digital sequencing approach for the detection of very low abundance ctDNA. At a timepoint similar to our T4, they reported that ctDNA was detected in 12 of 13 patients without pCR and in 5 of 9 patients with pCR^19^. Our analogous numbers are 13 of 17 without pCR and 2 of 7 with pCR. In addition, in our series, the absence of detectable ctDNA at this time point was predictive of relapse free survival and overall survival. Although we did not have sufficient patients to undertake multivariate analysis or to prognosticate non-pCR patients, our results show that the absence of ctDNA after NAC is at least similar to pCR or RCB score as a predictor of disease relapse (NPV for relapse-free survival of 89% for no ctDNA detection vs 86% for pCR). In our series, one patient with non-detectable ctDNA at the T4 time point suffered a disease relapse (Neo08). It is noteworthy that in this patient, we were only able to test one variant, as opposed to the average of 5 variants tested for all other patients.

In summary, our findings lead us to conclude that in cancers such as TNBCs, in which there are no frequently recurrent driver mutations except for TP53, probably at least 4–5 variants are required to obtain a sufficiently high sensitivity of detection and association with tumor features at baseline. After completion of NAC, we observed that a slight rise in ctDNA levels was predictive of incomplete pathological tumor response, but more importantly, the absence of ctDNA levels at this pre-surgical stage is associated with long-term relapse-free and overall survival, with a prognostic value similar to that of the RCB score or pCR. These results set the foundation for the testing of ctDNA in the post-NAC setting as a potential predictor of benefit from post-neoadjuvant chemotherapy administration, perhaps complimentary to RCB score. Indeed, the prognostic and predictive value of ctDNA as measured using a non-targeted, personalized approach, is evident in TNBC patients undergoing NAC, and has the potential to be used in their clinical management.

## Methods

### Patients

Triple-negative breast cancer patients undergoing NAC were recruited to the Quebec Clinical Research Organization in Cancer-03 trial (Q-CROC-03 trial, NCT01276899) from August 2010 to December 2013 in six centers in Canada and one site in the United States. All participants provided informed consent. The study was approved by the Ethics Committee Review Board of each participating institution (Comité d'éthique de la recherche du CHUM, Cook Country Health's Institutional Review Board, Research Ethics Boards of the McGill University Health Center, Comité d'éthique de la recherche de l'HSCM, Comité d'éthique de la recherche du Centre hospitalier affilié universitaire de Québec, The Research Ethics Board of Sunnybrook Health Sciences and Jewish General Hospital Research Ethics Office) and all research complied with local ethics guidelines. Of the entire cohort of 60 patients, tissue and serial blood samples for 26 patients were analyzed in this study (Supplementary Table S1). The reasons for the exclusions of the other 34 patients are illustrated in the diagram in Supplementary Fig. S1.

### Tumor samples sequencing

Sample collection and processing for this cohort have been detailed in Aguilar-Mahecha et al.^14^ In summary, tumor tissue was collected prior to chemotherapy at the time of the diagnostic biopsy (B1), and prior to surgery (serial biopsy) (B2) or during the surgical procedure (S3) if residual tumor was left after NAC (Fig. 1). The residual cancer burden (RCB) index was determined as previously reported by Symmans et al.^2^ DNA was extracted from tumor tissues as per reported SOPs^14^. DNA from frozen tumor samples and matched lymphocytes underwent WES and mutation calling at the McGill University Genome Quebec Innovation Center. A minimum of 500 ng of DNA was used to generate DNA libraries using Agilent’s SureSelect protocol as per manufacturer’s instructions. Neo42 and Neo28 had very low quantities of DNA extracted and libraries were therefore generated using the Nextera DNA library protocol (Illumina Inc.). We sequenced DNA from matched lymphocytes to use as a control for germline variants except for Neo30 where the patient’s normal breast tissue was used. WES was performed on Illumina HiSeq2000 platforms with 100 base paired-end reads. High-quality trimmed reads were aligned to the human reference genome (UCSC hg19) using the Burrows–Wheeler Alignment tool (BWA 0.7.12). Insertions/deletions (indels) were re-aligned using Genome Analysis Tool Kit (GATK). PCR duplicates were marked with Picard. Single nucleotide variants (SNVs) and indels were called by means of the SAMtools software. At the end, variants were annotated with ANNOVAR and custom in-house scripts.

### Blood collection and plasma processing

1. Q-CROC-03 patients.

Serial blood was collected prior to NAC and throughout the study until the time of surgery. The 1st collection was at baseline prior to treatment initiation (T0), the 2nd was after the 1st cycle of the 1st chemotherapy regimen (T1), the 3rd was at mid-treatment (T2), the 4th after the 1st cycle of the 2nd chemotherapy regimen (T3), and the last one at the end of chemotherapy, prior to surgery (T4) (Fig. 1 and Supplementary Table S1). For each time point, blood was collected in 2 × 4.5 ml BD Vacutainer CTAD Tubes and in 2 × 4 ml Vacutainer K_2_EDTA (Becton Dickinson) and centrifuged within 1 h of collection following SOPs. Samples were centrifuged at 1,500*g* for 15 min at room temperature (RT) and plasma immediately aliquoted and stored at − 80 °C. For the present study, aliquots were thawed and a second centrifugation at 16,000*g* was performed for 10 min at RT prior to analysis.

2. Healthy volunteer controls.

cfDNA of healthy control volunteers was used to set up a threshold of detection (ToD) for each allele frequency (AF) of all SNVs investigated. Blood from 30 healthy volunteers was collected by venipuncture in 2 × 4 ml BD Vacutainer K_2_EDTA tubes (Becton Dickinson) and immediately centrifuged at 1,500*g* for 10 min at RT. The plasma fraction was carefully collected and a second centrifugation at 16,000*g* was carried out at RT for 15 min. Plasma was aliquoted and stored at − 80 °C. All participants provided informed consent and the study was approved by the Jewish General Hospital (JGH) Ethics Committee Review Board and all research was performed in accordance with local ethics guidelines.

Plasma collected from these volunteers was then divided in three pools of 10 samples each and cfDNA extraction was performed from each of the 3 pooled samples. These 3 pools were used to obtain thresholds of detection for each ddPCR variant assay.

### cfDNA extraction

Extraction was performed from 3 ml of plasma using our previously reported protocol^15^. Components from both QIAamp DSP Kit (QIAGEN Cat# 61504) and QIAamp Circulating Nucleic Acid Kit (QIAGEN Cat# 55114) were used according to the following protocol: we added 125 μl QIAGEN Proteinase K/ ml of plasma and 1 ml of buffer AL (from DSP kit) containing carrier RNA (concentration of carrier RNA was established according to QIAGEN Circulating DNA kit handbook instructions) per 1 ml plasma. The lysate mixture was vortexed for 30 s and incubated at 56 °C for 15 min with occasional agitation. 1.2 ml of ACB buffer/ml of plasma was added to the lysate mixture, vortexed for 30 s and incubated for 5 min on ice. The lysate was applied onto the QIAamp MinElute column provided in the QIAGEN DSP kit and centrifugations were performed instead of using a vacuum pump. The lysate was applied in several elutions of 650 μl each followed by centrifugation at 10,000 rpm for 15 s, after applying the last 650 μl of lysate the column was centrifuged for 2 min. The flow-through from the collection tube was discarded and 600 μl of AW1 buffer (from DSP kit) was added to the column, incubated for 5 min at RT and then centrifuged at 10,000 rpm for one minute. After discarding the AW1 eluate, 750 μl of AW2 buffer (from DSP kit) was added to the column, incubated for 5 min at RT and then centrifuged at 14,000 rpm for two minutes. After the flow-through was discarded, 750 μl of EtOH (95–100%) was applied to the column, incubated for 5 min at RT and then spun at 14,000 rpm for 2 min. After the centrifugation the column was placed in a new collection tube, centrifuged for 3 min at 14,000 rpm and incubated at 56 °C for ten minutes to clear the column of interfering substances and improve the quality of DNA to be eluted in the next step. DNA elution consisted in the addition of 25 μl of AVE Buffer to the center of the membrane and after incubation at RT for 3 min the column was spun at 14,000 rpm for 2 min to collect the eluate, which was transferred to a clean 1.5 ml Eppendorf tube. The elution step was repeated twice. DNA was stored at − 20 °C for all the protocols tested for further ddPCR analysis.

### Development of ddPCR assays

SNVs previously identified by WES of the tumor were investigated by ddPCR. A set of primers and probes combination were designed for each SNV (Supplementary Table S3). Primers were analyzed for specificity using the electronic PCR (In-silico PCR) tool (https://genome.ucsc.edu/cgi-bin/hgPcr?command=start). ddPCR conditions were optimized to identify the optimal annealing temperature for each assay.

### Targeted pre-amplification

In order to increase the amount of DNA and test at least three SNVs in each patient sample, extracted cfDNA was used for targeted pre-amplification of each variant using primers and probes listed in Supplementary Table S3. We previously showed that targeted pre-amplification of cfDNA does not modify the fractional abundance of mutated variants in plasma, and does not alter the fractional abundance of ctDNA relative to wt DNA^15^.

cfDNA concentration was measured by QUBIT and 10 ng of cfDNA were used for targeted preamplification reactions with C-1000 touch thermal cycler (Bio-Rad) according to the manufacturer’s instructions. In order to test primers and probes for each SNV, 10 ng of tumor DNA from each patient was used for targeted pre-amplification and ddPCR analysis following protocols that we previously reported^15^.

### ddPCR variant calling and statistical analyses

Mutant Allele Frequency (MAF) was calculated as per standard ddPCR methods^15^ and represents the number of DNA fragments containing the specific mutated variant divided by the total amount (mutated and wild type) of cell free DNA (cfDNA) fragments. The *p* value for the MAF comparison according to the different demographical and tumor characteristics was calculated using a two-tailed Student’s *t* test when there were 2 independent samples and with an analysis of variance (ANOVA) test when there were more than 2 samples. We also used a two-tailed Student’s *t* test to compare MAF from different subgroups such as in pCR and non-pCR patients.

The comparison of number of pCR versus non-pCR patients for whom we detected SNVs was made with a Fisher’s exact test.

Relapse-free survival was defined as the time from surgery to relapse or last follow-up and overall survival as the time from diagnosis to death or last follow-up. Kaplan–Meier curves were constructed for RFS and OS using Graph Pad Prism (Version 8) and Log rank-proportional hazards analysis was performed to calculate unadjusted Hazard Ratios (HR) and 95% Confidence Intervals (IC). Log-rank (Mantel–Cox) test was used for statistical significance with *p* < 0.05 being significant.

## Supplementary information

Supplementary Information.

## Data Availability

Data will be made publicly available upon acceptance of the manuscript.
