# Supplementary material for: Prognostic and predictive value of circulating tumor DNA during neoadjuvant chemotherapy for triple negative breast cancer
Source: Sci Rep. 2020 Sep 7;10:14704. doi: 10.1038/s41598-020-71236-y (PMC7477566; doi:10.1038/s41598-020-71236-y)
Supplement: Supplementary file 1 — Supplementary Information. [file 41598_2020_71236_MOESM1_ESM.docx]

**Title: Prognostic and predictive value of circulating tumor DNA during neoadjuvant chemotherapy for triple negative breast cancer**

**Authors:** Luca Cavallone, Adriana Aguilar-Mahecha, Josiane Lafleur, Susie Brousse, Mohammed Aldamry, Talia Roseshter, Cathy Lan, Najmeh Alirezaie, Eric Bareke, Jacek Majewski, Cristiano Ferrario, Saima Hassan, Federico Discepola, Carole Seguin, Catalin Mihalcioiu, Elizabeth A. Marcus, André Robidoux, Josée-Anne Roy, Manuela Pelmus, Mark Basik

| **Time points for Blood samples** | **T0** | **T1** | **T2** | **T3** | **T4** |
| --- | --- | --- | --- | --- | --- |
| **Neo02** | At baseline | Post 1 AC | Drugs switch | Post 3 T | Post chemotherapy (=post 4AC+12T) |
| **Neo05** | At baseline | Post 1 T | - | **-** | Post chemotherapy (=post 9T) |
| **Neo06** | At baseline | Post 1 AC | Drugs switch | Post 3 T | At the end of chemotherapy (=post 4AC+11T (1T left)) |
| **Neo07** | - | Post 1 T | Post 3 T | Post 5T | Post chemotherapy (=post 12T) |
| **Neo08** | At baseline | Post 1 AC | Drugs switch | Post 3 T | At the end of chemotherapy (=post 4AC+11T (1T left)) |
| **Neo12** | At baseline | Post 2 AC | Drugs switch | Post 3 T | At the end of chemotherapy (=post 4AC+11T (1T left)) |
| **Neo14** | At baseline | Post 1 FEC | Drugs switch | Post 1Tr | At the end of chemotherapy (=post 3FEC+2Tr (1Tr left)) |
| **Neo17** | At baseline | Post 1 AC | Drugs switch | Post 3 T | At the end of chemotherapy (=post 4AC+11T (1T left)) |
| **Neo21** | At baseline | Post 1 AC | - | - | Post chemotherapy (=post 4AC+12T) |
| **Neo22** | At baseline | Post 1 EC | Drugs switch | Post 3 T | Post chemotherapy (=post 4EC+12T) |
| **Neo24** | At baseline | Post 1 T | Drugs switch | Post 1AC | At the end of chemotherapy (=post 12T+3AC (1AC left)) |
| **Neo25** | At baseline | Post 1 AC | Post 1 T | Post 3 T | At the end of chemotherapy (=post 4AC+11T (1T left)) |
| **Neo26** | At baseline | Post 1 AC | Drugs switch | Post 2 T | At the end of chemotherapy (=post 4AC+11T(1T left)) |
| **Neo27** | At baseline | Post 1 FEC | Drugs switch | Post 1Tr | At the end of chemotherapy (=post 3FEC+2Tr (1Tr left)) |
| **Neo28** | At baseline | Post 3 AC | Drugs switch | Post 3 T | Post chemotherapy (=post 4EC+7T) |
| **Neo30** | At baseline | Post 1 FEC | Drugs switch | Post 3 T | At the end of chemotherapy (=post 3FEC+11T (1T left)) |
| **Neo35** | At baseline | Post 1 FEC | Drugs switch | Post 3 T | At the end of chemotherapy (=post 3FEC+11T (1T left)) |
| **Neo38** | At baseline | Post 1 ACTr | Mid-treatment | Post 5ACTr | Post chemotherapy (=post 6ACTr) |
| **Neo39** | At baseline | Post 3 T | Mid-treatment | Post 7T | At the end of chemotherapy (=post 11T (1T left)) |
| **Neo40** | At baseline | - | - | - | At the end of chemotherapy (=post 4AC+4T (1T left)) |
| **Neo42** | At baseline | Post 1 ACTr | - | - | Post chemotherapy (=post 3ACTr) |
| **Neo44** | At baseline | Post 1 FEC | Drugs switch | Post 3 T | Post chemotherapy (=post 3FEC+12T) |
| **Neo49** | At baseline |  | Post 4AC | Post 2Tr | Post chemotherapy (=post 4AC&3Tr) |
| **Neo50** | At baseline | Post 1 T | Post 4 T | Post 5T | At the end of chemotherapy (=post 11T (1T left)) |
| **Neo57** | At baseline | Post 1 CarboT | Drugs switch | Post 2FEC | Post chemotherapy (=post 4Cartbo12T+3FEC) |
| **Neo58** | At baseline | Post 1 CarboT | Drugs switch | Post 1FEC | At the end of chemotherapy (=post 4Cartbo12T+2FEC+1EC (1EC left)) |

**Supplementary Table S1- Blood sample collection time points and drug regimen.** -: No sample collected; AC: Doxorubicin-Cyclophosphamide; ACTr: Doxorubicin-Cyclophosphamide- Docetaxel; CarboT: Carboplatin- Paclitaxel; EC: Epirubicin-Cyclophosphamide; FEC: 5-Fluorouracil-Epirubicin-Cyclophosphamide; T: Paclitaxel; Tr: Docetaxel.

| ***Patients*** | **Demographic characteristics** | | | **Tumor Characteristics pre-surgery** | | | | | | **Post-surgery***** | | | **Follow-up** | |
| --- | --- | --- | --- | --- | --- | --- | --- | --- | --- | --- | --- | --- | --- | --- |
|  | *Age at diagnosis (years)* | *Race* | *BRCA1/2 mutation** | *cT* | *cN* | *Clinical stage* | *Histological type*** | *Grade* | *ER, PR, Her2, FISH* | *pN* | *Residual tumor size (cm)* | *RCB Score* | *Relapse (month)* | *Death*  *(month)* |
| ***Neo02*** | 51.3 | Caucasian | NA | 2 | 1 | II | Ductal | 3 | 0,0,0,NA | Positive | > 2 | 3 | 1.17 | 14.9 |
| ***Neo05*** | 57.2 | Caucasian | NA | 2 | 1 | II | Ductal | 3 | <1,<1,0,NA | Positive | > 2 | 3 | 7.7 | 14.8 |
| ***Neo06*** | 52.9 | Caucasian | NA | 2 | 0 | II | Ductal | 3 | 0,<1,0,NA | Negative | 0 | 0 | - | - |
| ***Neo07*** | 57.5 | Asian | Yes | 2 | 0 | II | Ductal | 2 | 0,0,0,NA | Negative | > 2 | 2 | - | - |
| ***Neo08*** | 31.8 | Caucasian | No | 2 | 1 | II | Ductal | 3 | 0,<1,2+,1.1 | Negative | 0 | 0 | 11.0 | 21.2 |
| ***Neo12*** | 33.3 | Caucasian | Yes | 2 | 0 | II | Ductal | 3 | 0,0,2+,1 | Negative | 0 | 0 | - | - |
| ***Neo14*** | 68.5 | Caucasian | NA | 3 | 1 | III | Ductal | 3 | <1,<1,0,NA | Negative | 0 | 0 | - | - |
| ***Neo17*** | 50.4 | Caucasian | NA | 4 | 3 | III | Both types | 3 | <1,0,1+,1.1 | Positive | > 2 | 3 | 2.6 | 12.6 |
| ***Neo21*** | 39.9 | Caucasian | NA | 3 | 2 | III | Ductal | 3 | <1,<1,1+,1.2 | Negative | ≤ 2 | 1 | 19.0 | - |
| ***Neo22*** | 51.3 | Black | NA | 1 | 0 | I | Ductal | 3 | 0,0,1+,NA | Negative | 0 | 0 | - | - |
| ***Neo24*** | 63.5 | Caucasian | NA | 1 | 1 | II | Ductal | 3 | <1,<1,1+,0.9 | Positive | ≤ 2 | 3 | 19.6 | 52.9 |
| ***Neo25*** | 45.4 | Caucasian | NA | 2 | 0 | II | Ductal | 3 | <1,<1,1+,1.7 | Positive | > 2 | 3 | 13.1 | 24.1 |
| ***Neo26*** | 36.8 | Other | NA | 2 | 1 | II | Ductal | 3 | 0,0,1+,NA | Positive | 0 | 1 | - | - |
| ***Neo27*** | 40.5 | Caucasian | NA | 2 | 0 | II | Ductal | 2 | 0,0,2+, 1.6 | Negative | > 2 | 2 | 23.2 | 34.8 |
| ***Neo28*** | 52.9 | Caucasian | NA | 2 | 0 | II | Ductal | 2 | 5,<1,2+,1 | Negative | ≤ 2 | 2 | - | - |
| ***Neo30*** | 65.3 | Caucasian | NA | 2 | 1 | II | Ductal | 3 | 0,0,1+,NA | NA | NA | 3 | 1.0 | 20.6 |
| ***Neo35*** | 49.7 | Caucasian | NA | 2 | 0 | II | Ductal | 3 | 0,0,1+,NA | Negative | > 2 | 2 | - | - |
| ***Neo38*** | 49.4 | Black | NA | 4 | 1 | III | Ductal | 3 | 0,3,1+,1.1 | Positive | > 2 | 3 | 4.0 | 12.7 |
| ***Neo39*** | 82.0 | Caucasian | NA | 2 | 0 | II | Ductal | 3 | 0,0,1+,NA | Negative | > 2 | 2 | 28.7 | - |
| ***Neo40*** | 41.7 | Black | Yes | 2 | 0 | II | Ductal | 3 | 0,0,0,NA | Negative | 0 | 0 | - | - |
| ***Neo42*** | 43.9 | Caucasian | No | 3 | 2 | III | Lobular | 2 | 0,0,0,NA | Positive | > 2 | 3 | 14.2 | 24.4 |
| ***Neo44*** | 47.7 | Caucasian | NA | 2 | 0 | II | Ductal | 3 | <1,<1,1+,NA | Negative | > 2 | 2 | - | - |
| ***Neo49*** | 48.4 | Black | NA | 2 | 2 | III | Ductal | 3 | <1,0,2+,1.6 | Negative | 0 | 0 | - | - |
| ***Neo50*** | 47.6 | Caucasian | No | 2 | 0 | II | Both types | 2 | 0,0,2+,1.3 | Negative | > 2 | 2 | - | - |
| ***Neo57*** | 32.6 | Asian | Yes | 2 | 0 | II | Ductal | 3 | <1,5,0,NA | Negative | ≤ 2 | 1 | - | - |
| ***Neo58*** | 33.5 | Caucasian | NA | 2 | 1 | II | Ductal | 3 | 0,0,1+,NA | Negative | 0 cm | 0 | - | - |
| Total (n)/  Median | **48.9** | Cauc.: **19** Black: **4** Asian: **2** Hisp.: **0** Other: **1** | Yes **4**  No: **3** | 0: **2**  1: **19**  2: **3**  3: **2** | 0: **13**  1: **9**  2: **3**  3: **1** | I: **1**  II: **19**  III: **6** | Ductal: **25**  Lobular: **3** | 1: **0**  2: **5**  3: **21** |  | Neg: **17**  Pos: **8** | 0 cm: **9**  ≤ 2 cm: **4**  > 2 cm: **12** | 0: **8**  1: **3**  2: **7**  3: **8** | **12.1** | **20.9** |
| Percent. (%) / Average | **49.0** | Cauc.: **73** Black: **15** Asian: **8** Hisp.: **0** Other: **4** | Yes: **57**  No: **43** | 0: **8**  1: **73**  2: **12**  3: **8** | 0: **50**  1: **35**  2: **12**  3: **4** | I: **4**  II: **73**  III: **23** | Ductal: **89**  Lobular: **11** | 1: **0**  2: **19**  3: **81** |  | Neg: **68**  Pos: **32** | 0 cm: **36**  ≤ 2 cm: **16**  > 2 cm: **48** | 0: **31**  1: **12**  2: **27**  3: **31** | **12.1** | **23.3** |

**Supplementary Table S2- Patient and tumor characteristics.** * BRCA1/2 status: n=7, NA= no data available for 19 patients. ** n=28 because 2 patients had lobular and ductal breast cancer. *** One patient had no surgery due to the development of metastatic disease, so we have n =25 at the time of surgery for pN and residual tumor size. This patient was assigned a RCB score =3. cT: clinical tumor size; cN: clinical nodal status; pN: pathological nodal status.

| ***Patients*** | **Gene** | **Wild type (WT)** | **MUT** | **Protein alteration** | **Chr. location** | **Fragment length (bp)** | **Primer Forward** | **Primer Reverse** | **Probe WT HEX** | **Probe Mut FAM** | **ddPCR temp (°C)** |
| --- | --- | --- | --- | --- | --- | --- | --- | --- | --- | --- | --- |
| ***Neo02*** | **UTS2R** | C | G | p.R209G | chr17:80,332,825-80,332,825 | 81 | GGTCCCAAGAGCCTGT | GATGCTGGTGGCGAAG | CACCGCGCCTA | CACGGCGCCTA | 59 |
|  | **TP53** | G | A | p.R81X | chr17:7,578,212 | 182 | AGGTCAAATAAGCAGCAGGAG | GGAAATTTGCGTGTGGAGTATTT | CACTATGTCGAAAAG | ACACTATGTCAAAAAGT | 59 |
|  | **ZMYND15** | C | T | p.R469W | chr17:4,647,312-4,647,312 | 78 | GGCTCATGGCAGGATTAC | CAGCGGGTAGGTGAGA | CCCGCCATG | CCCACCATGT | 55 |
| ***Neo05*** | **DHX40** | G | A | p.R3Q | chr17:57,643,040-57,643,040 | 120 | AGATCGGTGGACGTGCT | TCTTCCTGGAGGTCTCTTGAC | TCCCGGTTTCC | TGTCCCAGTTTC | 59 |
|  | **ROBO2** | G | C | p.G523R | chr3:77,612,365-77,612,365 | 105 | GAGTCTGGAGCAACAATCAGTA | GGACAAGGTGACACTGTTCTTA | TGCCAGGGCC | CTGCCACGGCCA | 59 |
|  | **PARK2** | A | C | p.F155V | chr6:161,990,410-161,990,410 | 91 | AGTCTGATGCAGCCTTTGAG | GCTGTCCCAACTCCTTGATT | TCCTGAAGTGATGG | ATCCTGACGTGATG | 59 |
|  | **TRPM3** | G | T | p.L916M | chr9:73,168,179-73,168,179 | 76 | GATCCAAGCTCCTGTCTTG | TCCCTGTGGACAGAATGA | AGGCAGCTGGAT | AGGCATCTGGAT | 56 |
|  | **WIF1** | T | G | p.E367A | chr12:65,445,169-65,445,169 | 76 | GTCGGAGTTCACCAGATGTA | AGCACACGCCTTCACTTA | TCCTCGGCCTT | TCCGCGGCCT | 56 |
|  | **INSM1** | G | T | p.G242W | chr20:20,349,635-20,349,635 | 65 | AAGCTGCACTTCGAGGA | GGGCCCTCCTTGATCTT | TGCTGGGGCT | TGCTGTGGCT | 55 |
|  | **HDAC9** | AG | A | p.E670fs | chr7:18,788,734-18,788,735 | 86 | CATGCTGGACGAATACAGAG | TGTGTCCAATTCTGGATTACC | TGCAAGAAACTG | ACTGCAAAAACTGG | 55 |
|  | **ARID1A** | TGGCGGG | TGG | p.R1671fs | chr1:27,102,083-27,102,086 | 95/91 | AGACGACATGGAGGTTTATTTCA | TAATGCCCATGTGCTCTCTG | CATGGCGGGTAA | AGGCATGGTAATG | 56 |
|  | **KIF13B** | C | T | p.R730Q | chr8:28,999,819-28,999,819 | 108 | CCAAAGACCAAATCTGCTTTCC | TGTGTTTCTCACGGTCATTCT | AGCCTCGCTG | AGCCTTGCTGC | 56 |
|  | **CIT** | G | C | p.1136G | chr12:120,166,366-120,166,366 | 104 | CTTCCAGAAGCCTCTGTTTGA | GCTCAATGACCTGGAGAAGAA | AGCTTCGGGCAT | AGCTTCCGGCAT | 56 |
| ***Neo06*** | **DNAH9** | C | T | p.T3487M | chr17:11778483-11778483 | 96 | CCTCAGCTACAAGGCATCAA | TCTGTGCTCAACCACTTACC | TCACGCAGATTGGT | CGGGTCATGCA | 59 |
|  | **RAET1G** | C | T | p.G67S | chr6:150,240,839-150,240,839 | 100 | GCCGTTGTGACATTTAGTTTCT | AAGGCCAGGTGGATGAAA | ACTGTGGCAG | ACTGTAGCAGC | 56 |
|  | **HIST1H3C** | T | A | p.V47E | chr6:26,045,778-26,045,778 | 77 | TGAAGAAACCTCATCGCTACC | CAGCTCGGTGGACTTCTG | CACCGTGGC | CACCGAGGC | 56 |
|  | **TP53** | A | G | p.I63T | chr17:7,578,265-7,578,265 | 89 | TGTCATCCAAATACTCCACAC | GATTCCTCACTGATTGCTCTTA | CACTCGGATAAGA | CACTCGGGTA | 56 |
| ***Neo07*** | **PFKM** | A | G | p.N381S | chr12:48533646-48533646 | 87 | CCTGAAGACACCTCTCTCTATTTG | GGTCTGACATGAGCTAGAAGC | AACAACTGGGA | AACAGCTGGG | 54 |
|  | **GNA13** | G | A | p.R165X | chr17:63010731-63010731 | 90 | CCCGGTTATTGACGATTGTTTC | GTGAATTTGACCAGGTGCTTA | TCAGTCGATCTT | TCAGTCAATCTTCC | 59 |
|  | **NFKB1** | C | G | p.P771R | chr4:103531819-103531819 | 84 | AAATGCAGGAGAGGATGAAGG | GATGGAGCGGCACTCAC | TGTGCCTGGAA | TGTGCGTGGAA | 52 |
|  | **TP53** | A | G | p.F138S | chr17:7577129-7577129 | 92 | CCAGGACAGGCACAAACA | CTTACTGCCTCTTGCTTCTCTT | ACCTCAAAGCTGT | CCTCAGAGCTG | 52 |
| ***Neo08*** | **FLCN** | G | C | p.C11W | chr17:17131419-17131419 | 85 | ACCTCCGTGCAGAAGAGA | GTCTCCAAGGCACCATGAAT | CTCGCAGAAGT | CTCCCAGAAG | 57 |
| ***Neo12*** | **SACS** | CTTT | C | p.L574fs | chr13:23,927,943-23,927,946 | 95 | ACTAAACTATAATACCTCGGGTTTGG | TCCCAAGTCTTGAAGGAAGATTTAT | CAGCTTCCTTTAAA | CAGCTTCAAAGCA | 57 |
|  | **MRPL2** | CTTGGTCTCCT | AC | p.E115fs | chr6:43,024,095-43,024,105 | 85 | AACTTGCCTACAGGGATCATAG | ATGATTGACTTTCTGCGTTTCC | CTTGGTCTCCT | TCCTGACAGGC | 55 |
|  | **MROH7** | C | T | p.R711X | chr1:55172120-55172120 | 100 | TGGGCAATTGGTTCCTCTG | GCTCTGGCTGAGGAATATGAAG | CCACCGAGAC | CCACTGAGACA | 60 |
|  | **COL27A1** | G | C | p.R654P | chr9:116940623-116940623 | 81 | TACCTGGGCTACCTGGAAT | AGCCACCAAGAGTTCCAAAG | CCTCGGGTGA | CCTCCGGTGA | 55 |
| ***Neo14*** | **HTR5A** | G | A | p.R139H | chr7:154863025-154863025 | 92 | GGAACGTGACGGCCATAG | GACGTTGGAGACGCACTT | AGTAGCGGTCC | AGTAGTGGTCC | 57 |
|  | **TP53** | G | A | p.R116W | chr17:7577539-7577539 | 80 | GGAGTCTTCCAGTGTGATGATG | ACAACTACATGTGTAACAGTTCCT | CTCCGGTTCA | CTCCAGTTCA | 57 |
|  | **RB1** | ATTT | G | p.D527fs | chr13:48955463-48,955,468 | 82 | CCATGGATTCTGAATGTGCTTAAT | GTCAAGTTGCCTTCTGCTTTG | AGCCTTTGATTTTTAC | AGCCTTTGTTACAA | 55 |
| ***Neo 17*** | **TP53** | GGGCAGGTCTTGGCCAGTTGGCAAAACATCTTGT | G | frameshift deletion | chr17:7578505-7578505 | 153 | GTGCTGTGACTGCTTGTAGAT | TCTGTCTCCTTCCTCTTCCTAC | AGGTCTTGGCCAGTTGGCAAA | CAGCTGCACAGTGAGGGCAG | 60 |
|  | **HLF** | C | G | p.S258W | chr17:53398125-53398125 | 84 | GAGGCTGAAAGAGAACCAGAT | AAGTCAGCCACCTCCTG | CGGGCCTCGTTC | CGGGCCTGGTTC | 52 |
|  | **SALL3** | G | A | p.A386T | chr18:76753147-76753147 | 104 | CCGCTTCTACCTCAGACTT | TTCATGAGCGCGGACA | CCAGAGCGTTGG | CCAGAGTGTTG | 60 |
|  | **TNC** | C | T | p.D1888N | chr9:117797608-117797608 | 102 | CTGAACCTCAGTAGCAGTCAAG | CCCGAATTTCTGCCCTACAT | AGAATCGAGGT | AGAATTGAGGTC | 52 |
|  | **PKP3** | G | T | p.E551X | chr11:400619-400619 | 103 | GCCTCTACGACGAGATGC | GTGAAGCAGCCCACGA | CGGCTGGAGGG | CGGCTGTAGGG | 58 |
| ***Neo21*** | **ARFRP1** | T | G | p.N27H | chr20:62338365-62338365 | 94 | GGGCACTCACCGTCTTC | CTGCTGTCGGGCTTGTA | AGCATTGTCCA | AGCATGGTCCA | 52 |
|  | **CNTN6** | C | G | p.N418K | chr3:1,394,113-1,394,113 | 110 | CATGCATAGCCACAAATCAGTTT | GCCTGCATATTTACAATGTTCATCA | AAGAACACTGGC | AAGAAGACTGGC | 56 |
|  | **TRIM72** | C | T | p.P300L | chr16:31,235,541-31,235,541 | 112 | CAGCGAGGCAGAAACCT | GCCAGAGGAAGACACCAC | ACCCGAGCCT | CACCTGAGCCT | 59 |
|  | **TP53** | G | A | p.R150W | chr17:7,577,094-7,577,094 | 90 | CTTGCGGAGATTCTCTTCCT | GGTAATCTACTGGGACGGAAC | AGAGACCGGC | AGAGACTGGC | 59 |
| ***Neo22*** | **GCN1L1** | G | A | p.P1032L | chr12:120594789-120594789 | 84 | CCGATCACCCAAGTCAGAAG | CGACTTACCTGCCGTTCTC | TCCGGGCCAT | TCCAGGCCAT | 59 |
|  | **TP53** | A | C | p.L62R | chr17:7,578,268-7,578,268 | 97 | TCTGTCATCCAAATACTCCACAC | GCCTCTGATTCCTCACTGATT | TCGGATAAGATGCT | TCGGATACGATGC | 59 |
|  | **RAB21** | C | G | p.T33R | chr12:72149007-72149007 | 100 | GTTCAAGGTGGTGCTGCT | CAGAGTGGTGATGTGCTTGT | AGACGTCGCTGG | AGAGGTCGCTGG | 59 |
|  | **DHRS3** | A | C | p.C294G | chr1:12628398-12628398 | 86 | GCATGTCTTCATCCTGTCTCTAT | ACTCGAGGAGATCCACAAATTC | ATGCAGGTGT | CATGCCGGTGT | 55 |
|  | **PDZRN4** | G | C | p.K155N | chr12:41946493-41946493 | 135 | GTCATCTGTGTGTGTTGTTCTTT | CGTTTCTTACCTCGCTGACATA | AGAAGCTGGG | AGAACCTGGG | 55 |
| ***Neo24*** | **MTX2** | G | T | p.L53F | chr2:177188143-177188143 | 90 | ACTGGGCTTATTGTATGTATCTTGA | ATTCTGCATTTGCCCTACAAAC | ACTTGCCTATCA | AACTTTCCTATCA | 57 |
|  | **TP53** | C | G | p.A27P | chr17:7578455-7578455 | 77 | GTGCTGTGACTGCTTGTAGA | TGCAGCTGTGGGTTGATT | CCGCGCCATG | CCGCCCCATG | 57 |
|  | **ADCK4** | G | A | p.P290S | chr19:41206259-41206259 | 92 | CTTAGGCCCTGGCACTG | GTCCCAGCCGTGGTTAAG | TCCCCCTGGA | TCTCCCTGGA | 62 |
|  | **AOC3** | C | T | p.R153W | chr17:41003817-41003817 | 98 | CTTCTTTGGCAGGCAACC | CATGACGCTCCACAGTCA | CATGCGGGAC | CATGTGGGAC | 62 |
| ***Neo25*** | **GRM7** | C | T | p.A888V | chr3:7721947-7721947 | 93 | CCATGTCATCGAGGCTGT | ACTTACTGTTTGGGTCTACGTT | TTTGCCTCAC | CTTTACCTCACC | 57 |
|  | **ALKBH5** | G | A | p.R369H | chr17:18111631-18111631 | 100 | GGTAGCTTCAGCTCTGAGAA | GCCGCATCTTCACCTTTC | TTGCGCCAGT | TTGTGCCAGT | 59 |
|  | **PLXNA3** | A | G | p.M151V | chrX:153688974-153688974 | 100 | AAGGAGCACTACCTGTCG | CAGCAGTGCCCACAAAC | CTCCATGGCTG | CTCCGTGGCT | 59 |
|  | **COL14A1** | G | A | p.G1498E | chr8:121326208-121326208 | 98 | TACTTCTCTCTTGCCTTCCAG | AGACCACTTGGTCCTTGAG | CAGGACCTCA | CCAGAACCTCA | 53 |
|  | **TMPRSS2** | C | G | p.G134A | chr21:42861469-42861469 | 89 | CTCCCTGCACTTACTGAACT | CACTGTGCATCACCTTGAC | AGCTCCCACG | AGCTGCCACGA | 53 |
|  | **TP53** |  |  | p.R81X | chr17:7,578,212 | 182 | AGGTCAAATAAGCAGCAGGAG | GGAAATTTGCGTGTGGAGTATTT | CACTATGTCGAAAAG | ACACTATGTCAAAAAGT | 59 |
| ***Neo26*** | **PDE4C** | C | T | p.R105Q | chr19:18332966-18332966 | 66 | CCAGACTCACAGGTCGC | GACTATGAACTCTCGCCCAAG | TCTCGGAACT | TGTCTCAGAACT | 59 |
|  | **CNTNAP5** | C | A | p.Q1294K | chr2:125,671,824-125,671,824 | 87 | TGGACAGTTCCTTCAGAAATGA | ACCCTGCAGTTTCTCAGATG | TGACTTGCAAAAC | ACTTGAAAAACACA | 54 |
|  | **TP53** | G | A | p.R150W | chr17:7,577,094-7,577,094 | 85 | CGGAGATTCTCTTCCTCTGTG | GTAATCTACTGGGACGGAACAG | AGAGACCGGC | AGAGACTGGC | 52 |
|  | **SMC3** | G | C | p.G666A | chr10:112356189-112356189 | 78 | TTTGTTTATAGGTGACCAAGTCAG | TCAAGTCGAGACTTCCTTGTG | AACCCCCAGT | AACCCGCAGTT | 55 |
| ***Neo27*** | **TP53** | C | G | p.V41M | chr17:7578413-7578413 | 90 | CTGCTCACCATCGCTATCTG | ATGGCCATCTACAAGCAGTC | CCTCACAACCTC | CCTCAGAACCTC | 59 |
|  | **ROBO3** | G | T | p.W662C | chr11:124,744,718-124,744,718 | 91 | GGGTTCCTTGGTCAACAGATA | CTTCCGCCAGTCCCT | CCATGGAGAGGC | CATGTAGAGGC | 56 |
|  | **PRRG3** | G | A | p.R34Q | chrX:150868561-150868561 | 100 | GATTCCCTCGTGCCAATGA | ACTTCCTTGACCTCCTCGTA | ACTCTCGCTCG | ACTCTTGCTCG | 57 |
|  | **NOL7** | C | G | p.R5G | chr6:13615603-13615603 | 192 | AGGTCAGACGGTCTAGCG | CTTCCTCGTCTTCCTCCAG | CAGCTCCGAC | CAGCTCGGAC | 57 |
| ***Neo28*** | **SMARCA2** | C | T | p.R1213W | chr9:2116002-2116002 | 95 | GCATGTTTGACCAAAAGTCTTCA | GGTTTTCTCTAATACCTCATTTTCCT | CGAGCGGAGG | ACGAGTGGAGG | 55 |
|  | **GON4L** | G | A | p.R1639X | chr1:155730429-155730429 | 77 | ACTTGCCAGGGATATGTTGTA | GCCCAAATAAAGGGTGTTGAT | AGGTGCGAGAA | AGGTGTGAGAA | 55 |
|  | **SPTLC2** | T | A | p.N262I | chr14:78028804-78028804 | 80 | GATTCTAATGGTTGCTCCTGAC | GTTGCCTGATTCTGAGTGATG | TGCATGGTTCAGT | TGCATGGATCAGT | 59 |
|  | **PLEKHH1** | G | A | p.D1046N | chr14:68046546-68046546 | 93 | GAGAAAGCCATCCCACTCT | GTCACCTTGACACTTCCCT | CGGACGATCC | CGGACAATCCC | 52 |
| ***Neo30*** | **N4BP2L2** | C | T | p.D114N | chr13:33110825-33110825 | 82 | GGGTTTGTAAATGGGTCCT | GGAAGCACGTCCTCCAT | CTCATCGTCTGC | CTCATTGTCTGC | 57 |
|  | **PSMD1** | A | T | p.D609V | chr2:231951838-231951838 | 100 | TCTCATGTCAGTTTGGTCTAGTG | CTGCCCTCCTGACATCATC | TAACATCACTTACCT | TAACAACACTTACCT | 57 |
|  | **RP1** | A | T | p.N1385I | chr8:55540596-55540596 | 98 | GGTGATGACATTCAGAAAGATCTA | CTTAAATTACTGACATTTTGATGTGAC | CCAATGTATTAAATCC | CCAATGTATAAAATCC | 57 |
|  | **HIST1H1B** | T | G | p.K122N | chr6:27834942-27834942 | 99 | CGCGGGCTTCTTAGCTTTA | GGTGCTTCTGGCTCCTTTA | TTGGCTTTGGG | TTGGCGTTGGG | 57 |
|  | **PDE8B** | G | A | p.R339H | chr5:76703224-76703224 | 88 | GACACAGCTTTCTTGCCTTCT | TCGATGGTCATGGAGTGG | AGAATCGTCGC | CAGAATCATCGCTA | 57 |
|  | **MAGI2** | C | T | p.S4N | chr7:79082626-79082626 | 91 | CACTCTCATGGACTTTGCTAGT | AGGAGCCCAGGAACTGA | TGTCCAAAAGCT | ATGTCCAAAAACTTGA | 57 |
|  | **TP53** | CGG | CG | p.I200fs | chr17:7574029-7574029 | 105 | TGAGTTCCAAGGCCTCATTC | CAGGTACTGTGTATATACTTACTTCTCC | CCCACGGATCT | CCCACGATCTG | 59 |
| ***Neo35*** | **EVC2** | A | T | p.L562M | chr4:5627598-5627598 | 83 | ATTGTCCAACTTCTGCTTGATTG | GGTACCTGGATGAAGACCAAAT | CGCTCCAATAGC | CGCTCCATTAGC | 57 |
|  | **GALNT9** | A | T | p.V86E | chr12:132685715-132685715 | 75 | GTACGTGAGGGTGTTGTTGT | AAGTGTCGCAGCTTCAAGT | AACGTGTACCC | AACGAGTACCC | 57 |
|  | **DCX** | G | A | p.R120X | chrX:110653512-110653512 | 100 | CCTTCTTGGCTTTCTTCTCATTAC | CGGATGAATGGGTTGCCTA | CTACCGAACCA | CTACTGAACCA | 55 |
|  | **TP53** | GCCCAGAC/GGAAACCG | GCCCAGAC/ggttt/GGAAACCG | p.F70fs | chr17:7579359-7579359 | 85/90 | ACTTGGCTGTCCCAGAATG | CTGTCATCTTCTGTCCCTTCC | CAGACGGAAAC | AGACGGTTTGGAAA | 57 |
| ***Neo38*** | **MAP7D3** | C | T | p.R576H | chrX:135310836-135310836 | 93 | GCATTTCTTCCCGTTGTCTTTC | CCGAGGCGGCAACAAA | TGTTCACGAGCAAG | TGTTCATGAGCAAG | 60 |
|  | **ROBO2** | C | T | p.T59M | chr3:77,147,279-77,147,279 | 93 | AGCCCACGACTCTGAAC | CGTCCTTGTCAGTCTCCA | CCAACGCCCACC | CCAATGCCCAC | 57 |
|  | **KIF19** | C | A | p.L280M | chr17:72,342,577-72,342,577 | 100 | AATCGTGGGCAGCGTATG | ACTTGTTGCTACCCTTGTCG | CTGGCACTGGGC | CTGGCAATGGGCA | 57 |
| ***Neo39*** | **TP53** | C | T | p.E153K | chr17:7,577,085-7,577,085 | 95 | CCCTTTCTTGCGGAGATTC | GTAATCTACTGGGACGGAACA | CGCACAGAGGA | CGCACAAAGGA | 57 |
|  | **DNAH7** | T | A | p.K345N | chr2:196,866,537-196,866,537 | 95 | AAT TTG GCA CTG CTG TCA | TTT GTT CTT TCA AGG TGG TTT C | AAATAAGCAATTGCCA | AAAAAAGCAATTGCCAA | 56 |
|  | **RASA1** | G | C | p.E338D | chr5:86633905-86633905 | 107 | GGCCTTATTGTTGAAGACCTAGTA | CTCTGATTAYGGACAAGATCCAA | AAGAGGTGGT | AAGACGTGGTA | 56 |
|  | **GRIN3A** | G | A | p.R.137C | chr9:104,499,853-104,499,853 | 75 | GGCCATCACTACTTCCAAAGAC | CTATTTGCCGTGGACAACCT | CACGCGGTTC | CACGCAGTTCA | 59 |
|  | **ROBO1** | C | A | p.R1421I | chr3:78,656,056-78,656,056 | 120 | GCATGTCAACAACCTGTCTTC | GACCTGTAGTGGTGCCAAA | TCCTTTTCTGTCTGA | TCCTTTTATGTCTGA | 57 |
|  | **MAP3K4** | T | C | NA | chr6:161,530,948-161,530,948 | 110 | CAGATCACCATTGCGATCAAC | TGGGTACCAGTGGCTCT | AAGGTAATCCC | AAGGCAATCCC | 52 |
|  | **UNC5C** | A | G | p.F45S | chr4:96,256,773-96,256,773 | 91 | CTCAGGTGGATCAGAAGGA | ATGTGTTAACCATCTTCCAC | CAGATGATGACTTTTTTC | CAGATGATGACTCTTT | 54 |
|  | **GRIA1** | G | A | p.G665S | chr5:153149938-153149938 | 95 | ACCATGAAGGTGGGAGGTA | AGGAAGCAAATCTTGGCTACTTA | CCAAAGGCTATG | TCCAAAAGCTATGG | 55 |
|  | **DNAH2** | C | T | p.R3387X | chr17:7721017-7721017 | 100 | ACGCCTTCTCCACTGAG | GAGGCCCTCTCTCCCAT | CACCCGAGGC | TCACCTGAGGC | 55 |
|  | **SLC25A4** | G | A | p.G102D | chr4:186066111-186066111 | 83 | AAGCTCTCAACTTCGCCTTC | GTAGCGCCAGAACTGCTTAT | TCCACACCCCC | TCCACATCCCC | 60 |
|  | **ACTL7B** | G | T | p.P69Q | chr9:111618005-111618005 | 96 | ACGGTGGAGGAGATGAAGTA | CAAGATCAAGGCGGTCATCA | CCTCGGCTCT | CCTCTGCTCTC | 55 |
|  | **SBF2** | G | T | p.S1243Y | chr11:9850968-9850968 | 84 | TGCTGTTGCCTCTGAGTTT | CTTCCAGTAGCATAGAACAAGAGA | TGGACAGAAACAG | TGGACATAAACAGC | 55 |
| ***Neo40*** | **PRDM15** | C | A | p.D53Y | chr21:43,279,786-43,279,786 | 100 | GAGTCTTTGACCATGACCACT | CTCTAATGCCCGGATGTCTG | TGCAGTCTTCAC | TGCAGTATTCACA | 57 |
|  | **AGBL1** | C | T | p.Q584X | chr15:86813199-86813199 | 93 | TGGTCAACGCAGATGTGAATA | TGTTGAAGTGGTAAGGGATGG | AGCACCAGCA | AGCACTAGCA | 52 |
|  | **TP53** | C | T | p.R116Q | chr17:7577538-7577538 | 80 | GGAGTCTTCCAGTGTGATGATG | ACAACTACATGTGTAACAGTTCCT | CCTCCGGTTC | CCTCTGGTTCA | 55 |
|  | **RB1** | C | T | p.Q395X | chr13:48,947,596-48,947,596 | 130 | GACTGTTATGAACACTATCCAACAAT | TGCCAAGATATTACAATAAATAATGTTTCA | AGGTTGATCAC | TGAAGGTTAATCACT | 54 |
| ***Neo42*** | **ZFP36L1** | T | C | p.H139R | chr14:69256851-69256851 | 97 | CAGCTCCGTCTTGTACTTGG | AAACGGTGCCTGTAAGTACG | CCGTGTGCGAA | CCGCGTGCGA | 55 |
|  | **FMN1** | T | A | p.D334V | chr15:33359085-33359085 | 85 | AATGTTGAGCAGCAGAGAGAG | GCAGACAGTGAAGAGTGAGAAG | TCGGATCAGTTG | CGGAACAGTTGC | 57 |
|  | **SLC16A2** | C | T | p.L494F | chrX:73751248-73751248 | 91 | CCTTCTACTTTGCCGGTGT | TGCTCTTTCTTGAACATCCTTTG | AGAGGATTACA | AAGAAGATTACAGC | 55 |
|  | **GNAT1** | G | A | p.E167K | chr3:50231235-50231235 | 97 | CAGCTACCTCTCCGACCT | GATGATGCCAGTGGTCTTGA | CCACCGAGCA | CCACCAAGCAGG | 55 |
|  | **TP53** | C | G | p.R116P | chr17:7577538-7577538 | 108 | GGAGTCTTCCAGTGTGATGATG | GTTGGCTCTGACTGTACCAC | CCTCCGGTTCA | CCTCGGGTTCA | 59 |
| ***Neo44*** | **ATR** |  | del of AACACAATTCAGGA | p.F1188fs | chr3:142,259,750-142,259,763 | 140 | ACCCAATTTCCCTCAAAGAGATA | GAGAACTGGCCTTCGATTCA | AACACAATTCAGGA | TACCTGCAAATCAT | 60 |
|  | **FGFR2** | T | G | p.E665D | chr10:123,239,506-123,239,506 | 88 | ACAGAATCATCTCCTGAAGAACAA | AATACTTGGACCTCAGCCAAC | ACTGTTCGAGAG | ACTGGTCGAGAG | 57 |
|  | **CASP8** | G | T | p.G403V | chr2:202,149,989-202,149,989 | 78 | CCACTGTGAATAACTGTGTTTCC | TCAGGCTCTGGCAAAGTG | AGGGAACCTG | AGGTAACCTG | 57 |
|  | **TP53** | A | T | p.L72Q | chr17:7,579,355-7,579,355 | 85 | ACTTGGCTGTCCCAGAATG | CTGTCATCTTCTGTCCCTTCC | AGCCCAGACG | AGCCCTGACG | 57 |
|  | **HPN** | G | A | p.D352N | chr19:35556775-35556775 | 79 | CAGCCAGACCTCCCTCT | GTGGCGTCCGAGAGATG | CGCTGTCGCC | CGCTGTTGCC | 55 |
| ***Neo49*** | **HIST1H2BF** | C | G | p.S37R | chr6:26199897-26199897 | 100 | AAAGGCGGTGACCAAGG | GACCTGCTTTAGCACCTTGTA | AGAGCTATTCCG | AGAGGTATTCCG | 57 |
|  | **HIST1H2BJ** | C | G | p.E114D | chr6:27100188-27100188 | 96 | TGTACTTGGTGACGGCCT | ACCTCCAGGGAGATCCAG | TACCCTCGGAC | TACCGTCGGAC | 57 |
|  | **TNFRSF18** | G | T | p.T119N | chr1:1139821-1139821 | 92 | CCACCCAGGACTCACTCT | TTTGGCTTCCAGTGTATCGAC | AGAAGGTCCCC | AGAAGTTCCCC | 57 |
|  | **TADA2A** | G | A | p.E136K | chr17:35800727-35800727 | 85 | TACCCTGCTGAACCTGAAAC | CAAGATAGGGAGGCACTTACAG | AGAGGAAGCA | AGCAGAGAAAGCA | 57 |
| ***Neo50*** | **EXOC4** | G | T | p.R872S | chr7:133692517-133692517 | 77 | ATCAGTGAGTCTGGCATCAA | GACATGGTGATGTTGGTCAAA | TGTGTAGGAACA | AAATGTGTAGTAACAT | 55 |
|  | **RNF123** | A | G | p.Y1218C | chr3:49758242-49758242 | 80 | TCCTCAGCTACCTCTGACTT | CACTTGGGCCAGCTCAT | TGCGGATTATATCA | CGGATTGTATCA | 57 |
|  | **GPR158** | C | T | p.T1015I | chr10:25887599-25887599 | 84 | GTGTGTCCTTGGGAGGTTTAT | AGCCACAATAGATACGTGCTT | CTGACCCCTG | CTGATCCCTGG | 53 |
|  | **TP53** | C | T | p.W14X | chr17:7579528-7579528 | 90 | ATTCTGGGAGCTTCATCTGG | GTCCCAAGCAATGGATGATTT | AATGGTTCACT | ACAATGATTCACTG | 52 |
| ***Neo57*** | **ANKRD12** | G | T | p.R1911L | chr18:9,275,559-9,275,559 | 142 | GGCGAGAGAGTAAGACTGTTC | CCAATGTTCTTGCAGCTCTG | TTGCGAGTTCA | TTGCTAGTTCATT | 57 |
|  | **NR5A1** | T | A | p.E304V | chr9:127,255,388-127,255,388 | 118 | GGGACTGGTCACCTCCT | GACGCTGCTGCAGAACT | CAGCTCGCTC | CAGCACGCTC | 57 |
|  | **BAP1** | G | C | p.H169Q | chr3:52,441,263-52,441,263 | 82 | GGCCTGTGATAGGCACATAG | CTCCCTGAGAAGCAGAATGG | TGACAAAGTGGA | TGACAAACTGGA | 52 |
|  | **TFCP2** | G | T | p.A24D | chr12:51566135-51566135 | 93 | ATGCTATAGGCACCAGCAC | GGCCGACGAAGTGATTGA | AGGCTAGCATCA | CAGGCTATCATCA | 57 |
| ***Neo58*** | **DNAJC12** | G | A | p.S84L | chr10:69,571,328-69,571,328 | 112 | CACCGTCTTCACTGAGTCATT | GGCAAAGGAGATTCTGACCAA | CATCGACATCT | TGGCATCAACATCT | 58 |
|  | **YEATS2** | G | T | p.K523N | chr3:183,476,666-183,476,666 | 78 | TTTAATTGTGCCTTGCTTATCAGG | GGAACCTGTTCCTTGGGAG | AACAAGATCTCC | CAAACAATATCTCCAC | 57 |
|  | **HIPK2** | deletion of ATTGGAGTGATTCTCTTG | ATTG | p.D513fs | chr7:139,311,411-139,311,424 | 94 | TGGTGACAAAGGGATGGTTC | CGGGAGTTCATTGACCTGTT | TTGGAGTGATTCTC | TTCGATTGTCAGC | 60 |
|  | **ZDHHC2** | A | G | p.N258S | chr8:17067483-17067483 | 99 | TCCAGTATTTCGACATGGAACAG | AGCAACCAGTACTTCTTCTCATC | AGAATGGATTCAGCT | AGAGTGGATTCA | 52 |
| **Average** | | | | | **Fragment length** | **95.28** | **Primer length** | **20.30** | **Probe length/**  **ddPCR Temp.** | **11.76** | **56.44** |
| **Standard Deviation** | | | | |  | **18.84** |  | **2.00** |  | **1.89** | **2.32** |
| **Median** | | | | |  | **92.50** |  | **20.00** |  | **11.00** | **57.00** |
| **Minimum** | | | | |  | **65.00** |  | **16.00** |  | **9.00** | **52.00** |
| **Maximum** | | | | |  | **192.00** |  | **28.00** |  | **20.50** | **62.00** |

**Supplementary Table S3- Variant identification with specific primers and probes for ddPCR.**

| ***Patients*** | ***Gene*** | ***MAF*** | | | | | | ***Threshold of Detection*** |
| --- | --- | --- | --- | --- | --- | --- | --- | --- |
|  |  | **Pool 1** | **Pool 2** | **Pool 3** | **Average** | **SD** | **2SD** |  |
| ***Neo02*** | **TP53** | 0.110 | 0.080 | 0.090 | 0.093 | 0.015 | 0.031 | **0.124** |
|  | **UTS2R** | 0.000 | 0.011 | 0.000 | 0.004 | 0.006 | 0.013 | **0.016** |
|  | **ZMYND15** | 0.060 | 0.043 | 0.061 | 0.055 | 0.010 | 0.020 | **0.075** |
| ***Neo05*** | **ARID1A** | 0.000 | 0.000 | 0.000 | 0.000 | 0.000 | 0.000 | **0.000** |
|  | **CIT** | 0.006 | 0.019 | 0.016 | 0.014 | 0.007 | 0.014 | **0.027** |
|  | **DHX40** | 0.019 | 0.040 | 0.017 | 0.025 | 0.013 | 0.025 | **0.051** |
|  | **HDAC9** | 0.045 | 0.030 | 0.071 | 0.049 | 0.021 | 0.041 | **0.090** |
|  | **INSM1** | 0.000 | 0.000 | 0.019 | 0.006 | 0.011 | 0.022 | **0.028** |
|  | **KIF13B** | 0.041 | 0.051 | 0.065 | 0.052 | 0.012 | 0.024 | **0.076** |
|  | **WIF1** | 0.011 | 0.000 | 0.000 | 0.004 | 0.006 | 0.013 | **0.016** |
| ***Neo06*** | **DNAH9** | 0.040 | 0.051 | 0.091 | 0.061 | 0.027 | 0.054 | **0.114** |
|  | **HIST1H3C** | 0.033 | 0.000 | 0.027 | 0.020 | 0.018 | 0.035 | **0.055** |
|  | **RAET1G** | 0.016 | 0.012 | 0.017 | 0.015 | 0.003 | 0.005 | **0.020** |
|  | **TP53** | 0.014 | 0.021 | 0.001 | 0.012 | 0.010 | 0.020 | **0.032** |
| ***Neo07*** | **GNA13** | 0.066 | 0.039 | 0.100 | 0.068 | 0.031 | 0.061 | **0.129** |
|  | **NFKB1** | 0.000 | 0.000 | 0.000 | 0.000 | 0.000 | 0.000 | **0.000** |
|  | **PFKM** | 0.073 | 0.037 | 0.070 | 0.060 | 0.020 | 0.040 | **0.100** |
|  | **TP53** | 0.126 | 0.290 | 0.094 | 0.170 | 0.105 | 0.210 | **0.380** |
| ***Neo08*** | **FLCN** | 0.023 | 0.000 | 0.000 | 0.008 | 0.013 | 0.027 | **0.034** |
| ***Neo12*** | **COL27A1** | 0.000 | 0.000 | 0.013 | 0.004 | 0.008 | 0.015 | **0.019** |
|  | **MROH7** | 0.040 | 0.015 | 0.036 | 0.030 | 0.013 | 0.027 | **0.057** |
| ***Neo14*** | **HTR5A** | 0.058 | 0.062 | 0.138 | 0.086 | 0.045 | 0.090 | **0.176** |
|  | **RB1** | 0.000 | 0.000 | 0.000 | 0.000 | 0.000 | 0.000 | **0.000** |
|  | **TP53** | 0.031 | 0.045 | 0.059 | 0.045 | 0.014 | 0.028 | **0.073** |
| ***Neo17*** | **HLF** | 0.000 | 0.000 | 0.000 | 0.000 | 0.000 | 0.000 | **0.000** |
|  | **PKP3** | 0.000 | 0.000 | 0.000 | 0.000 | 0.000 | 0.000 | **0.000** |
|  | **SALL3** | 0.050 | 0.040 | 0.060 | 0.050 | 0.010 | 0.020 | **0.070** |
|  | **TNC** | 0.004 | 0.025 | 0.036 | 0.022 | 0.016 | 0.033 | **0.054** |
| ***Neo21*** | **ARFRP1** | 0.000 | 0.000 | 0.000 | 0.000 | 0.000 | 0.000 | **0.000** |
|  | **CNTN6** | 0.000 | 0.000 | 0.007 | 0.002 | 0.004 | 0.008 | **0.010** |
|  | **TP53** | 0.060 | 0.050 | 0.080 | 0.063 | 0.015 | 0.031 | **0.094** |
|  | **TRIM72** | 0.090 | 0.046 | 0.078 | 0.071 | 0.023 | 0.045 | **0.117** |
| ***Neo22*** | **DHRS3** | 0.000 | 0.110 | 0.000 | 0.037 | 0.064 | 0.127 | **0.164** |
|  | **GCN1L1** | 0.082 | 0.100 | 0.080 | 0.087 | 0.011 | 0.022 | **0.109** |
|  | **PDZRN4** | 0.010 | 0.004 | 0.000 | 0.005 | 0.005 | 0.010 | **0.015** |
|  | **RAB21** | 0.011 | 0.011 | 0.011 | 0.011 | 0.000 | 0.000 | **0.011** |
|  | **TP53** | 0.032 | 0.022 | 0.014 | 0.023 | 0.009 | 0.018 | **0.041** |
| ***Neo24*** | **ADCK4** | 0.013 | 0.013 | 0.010 | 0.012 | 0.002 | 0.003 | **0.015** |
|  | **AOC3** | 0.077 | 0.089 | 0.073 | 0.080 | 0.008 | 0.017 | **0.096** |
|  | **MTX2** | 0.000 | 0.000 | 0.000 | 0.000 | 0.000 | 0.000 | **0.000** |
|  | **TP53** | 0.000 | 0.000 | 0.000 | 0.000 | 0.000 | 0.000 | **0.000** |
| ***Neo25*** | **ALKBH5** | 0.040 | 0.050 | 0.040 | 0.043 | 0.006 | 0.012 | **0.055** |
|  | **COL14A1** | 0.000 | 0.000 | 0.050 | 0.017 | 0.029 | 0.058 | **0.074** |
|  | **GRM7** | 0.046 | 0.015 | 0.035 | 0.032 | 0.016 | 0.031 | **0.063** |
|  | **PLXNA3** | 0.090 | 0.090 | 0.060 | 0.080 | 0.017 | 0.035 | **0.115** |
|  | **TMPRSS2** | 0.000 | 0.000 | 0.000 | 0.000 | 0.000 | 0.000 | **0.000** |
|  | **TP53** | 0.060 | 0.000 | 0.130 | 0.063 | 0.065 | 0.130 | **0.193** |
| ***Neo26*** | **CNTNAP5** | 0.018 | 0.002 | 0.007 | 0.009 | 0.008 | 0.016 | **0.025** |
|  | **PDE4C** | 0.059 | 0.084 | 0.117 | 0.087 | 0.029 | 0.058 | **0.145** |
|  | **TP53** | 0.056 | 0.075 | 0.049 | 0.060 | 0.013 | 0.027 | **0.087** |
| ***Neo27*** | **NOL7** | 0.000 | 0.000 | 0.000 | 0.000 | 0.000 | 0.000 | **0.000** |
|  | **PRRG3** | 0.055 | 0.090 | 0.052 | 0.066 | 0.021 | 0.042 | **0.108** |
|  | **ROBO3** | 0.000 | 0.003 | 0.000 | 0.001 | 0.001 | 0.003 | **0.004** |
|  | **TP53** | 0.000 | 0.000 | 0.000 | 0.000 | 0.000 | 0.000 | **0.000** |
| ***Neo28*** | **GON4L** | 0.014 | 0.071 | 0.053 | 0.046 | 0.029 | 0.058 | **0.104** |
|  | **PLEKHH1** | 0.016 | 0.025 | 0.022 | 0.021 | 0.005 | 0.009 | **0.030** |
|  | **SMARCA2** | 0.104 | 0.090 | 0.063 | 0.086 | 0.021 | 0.042 | **0.127** |
|  | **SPTLC2** | 0.018 | 0.013 | 0.014 | 0.015 | 0.003 | 0.005 | **0.020** |
| ***Neo30*** | **HIST1H1B** | 0.023 | 0.009 | 0.071 | 0.034 | 0.033 | 0.065 | **0.099** |
|  | **MAGI2** | 0.029 | 0.060 | 0.037 | 0.042 | 0.016 | 0.032 | **0.074** |
|  | **N4BP2L2** | 0.082 | 0.180 | 0.078 | 0.113 | 0.058 | 0.116 | **0.229** |
|  | **PDE8B** | 0.090 | 0.054 | 0.070 | 0.071 | 0.018 | 0.036 | **0.107** |
|  | **PSMD1** | 0.042 | 0.049 | 0.034 | 0.042 | 0.008 | 0.015 | **0.057** |
|  | **RP1** | 0.130 | 0.100 | 0.100 | 0.110 | 0.017 | 0.035 | **0.145** |
|  | **TP53** | 0.000 | 0.000 | 0.000 | 0.000 | 0.000 | 0.000 | **0.000** |
| ***Neo35*** | **DCX1** | 0.107 | 0.052 | 0.098 | 0.086 | 0.030 | 0.059 | **0.145** |
|  | **EVC2** | 0.160 | 0.012 | 0.017 | 0.063 | 0.084 | 0.168 | **0.231** |
|  | **GALNT9** | 0.012 | 0.034 | 0.068 | 0.038 | 0.028 | 0.056 | **0.094** |
|  | **TP53** | 0.000 | 0.000 | 0.000 | 0.000 | 0.000 | 0.000 | **0.000** |
| ***Neo38*** | **KIF19** | 0.000 | 0.090 | ? | 0.045 | 0.064 | 0.127 | **0.172** |
|  | **MAP7D3** | 0.090 | 0.041 | 0.113 | 0.081 | 0.037 | 0.074 | **0.155** |
|  | **ROBO2** | 0.052 | 0.070 | ? | 0.061 | 0.013 | 0.025 | **0.086** |
| ***Neo39*** | **ACTL7B** | 0.000 | 0.013 | 0.023 | 0.012 | 0.012 | 0.023 | **0.035** |
|  | **DNAH2** | 0.060 | 0.140 | 0.160 | 0.120 | 0.053 | 0.106 | **0.226** |
|  | **DNAH7** | 0.061 | 0.070 | 0.029 | 0.053 | 0.022 | 0.043 | **0.096** |
|  | **GRIA1** | 0.060 | 0.043 | 0.029 | 0.044 | 0.016 | 0.031 | **0.075** |
|  | **GRIN3A** | 0.059 | 0.110 | 0.130 | 0.100 | 0.037 | 0.073 | **0.173** |
|  | **MAP3K4** | 0.061 | 0.038 | 0.047 | 0.049 | 0.012 | 0.023 | **0.072** |
|  | **SLC25A4** | 0.029 | 0.040 | 0.005 | 0.025 | 0.018 | 0.036 | **0.060** |
|  | **TP53** | 0.040 | 0.027 | 0.022 | 0.030 | 0.009 | 0.019 | **0.048** |
|  | **UNC5C** | 0.280 | 0.280 | 0.320 | 0.293 | 0.023 | 0.046 | **0.340** |
| ***Neo40*** | **AGBL1** | 0.018 | 0.043 | 0.007 | 0.023 | 0.018 | 0.037 | **0.060** |
|  | **PRDM15** | 0.000 | 0.000 | 0.000 | 0.000 | 0.000 | 0.000 | **0.000** |
|  | **RB1** | 0.000 | 0.000 | 0.000 | 0.000 | 0.000 | 0.000 | **0.000** |
|  | **TP53** | 0.105 | 0.051 | 0.128 | 0.095 | 0.040 | 0.079 | **0.174** |
| ***Neo42*** | **FMN1** | 0.023 | 0.017 | 0.013 | 0.018 | 0.005 | 0.010 | **0.028** |
|  | **GNAT1** | 0.030 | 0.031 | 0.065 | 0.042 | 0.020 | 0.040 | **0.082** |
|  | **SLC16A2** | 0.045 | 0.013 | 0.020 | 0.026 | 0.017 | 0.034 | **0.060** |
|  | **TP53** | 0.006 | 0.004 | 0.003 | 0.004 | 0.002 | 0.003 | **0.008** |
|  | **ZFP36L1** | 0.109 | 0.086 | 0.095 | 0.097 | 0.012 | 0.023 | **0.120** |
| ***Neo44*** | **ATR** | 0.000 | 0.000 | 0.000 | 0.000 | 0.000 | 0.000 | **0.000** |
|  | **CASP8** | 0.015 | 0.000 | 0.000 | 0.005 | 0.009 | 0.017 | **0.022** |
|  | **FGFR2** | 0.054 | 0.011 | 0.005 | 0.023 | 0.027 | 0.053 | **0.077** |
|  | **HPN** | 0.098 | 0.055 | 0.072 | 0.075 | 0.022 | 0.043 | **0.118** |
|  | **TP53** | 0.114 | 0.112 | 0.061 | 0.096 | 0.030 | 0.060 | **0.156** |
| ***Neo49*** | **TADA2A** | 0.030 | 0.040 | 0.018 | 0.029 | 0.011 | 0.022 | **0.051** |
|  | **TNFRSF18** | 0.000 | 0.000 | 0.000 | 0.000 | 0.000 | 0.000 | **0.000** |
| ***Neo50*** | **EXOC4** | 0.003 | 0.014 | 0.009 | 0.009 | 0.006 | 0.011 | **0.020** |
|  | **GPR158** | 0.007 | 0.016 | 0.000 | 0.008 | 0.008 | 0.016 | **0.024** |
|  | **RNF123** | 0.024 | 0.067 | 0.036 | 0.042 | 0.022 | 0.044 | **0.087** |
|  | **TP53** | 0.130 | 0.170 | 0.120 | 0.140 | 0.026 | 0.053 | **0.193** |
| ***Neo57*** | **ANKRD12** | 0.008 | 0.000 | 0.000 | 0.003 | 0.005 | 0.009 | **0.012** |
|  | **BAP1** | 0.000 | 0.000 | 0.000 | 0.000 | 0.000 | 0.000 | **0.000** |
|  | **NR5A1** | 0.024 | 0.019 | 0.140 | 0.061 | 0.068 | 0.137 | **0.198** |
|  | **TFCP2** | 0.015 | 0.019 | 0.022 | 0.019 | 0.004 | 0.007 | **0.026** |
| ***Neo58*** | **DNAJC12** | 0.113 | 0.053 | 0.065 | 0.077 | 0.032 | 0.063 | **0.140** |
|  | **HIPK2** | 0.000 | 0.001 | 0.000 | 0.000 | 0.001 | 0.002 | **0.002** |
|  | **YEATS2** | 0.000 | 0.007 | 0.000 | 0.002 | 0.004 | 0.008 | **0.010** |
|  | **ZDHHC2** | 0.127 | 0.110 | 0.170 | 0.136 | 0.031 | 0.062 | **0.198** |

**Supplementary Table S4- MAF in normal control plasma and threshold of detection derived for each detectable variant.** SD: standard deviation

| ***Patients*** | ***Gene*** | ***Variation*** | ***Position*** | ***Ref*** | ***Alt*** | ***Protein Change*** | ***CADDa*** | ***Pre-NAC***  ***Tumor MAF*** | | ***Post-NAC***  ***Tumor MAF*** | ***T0*** | ***T1*** | ***T2*** | ***T3*** | ***T4*** |
| --- | --- | --- | --- | --- | --- | --- | --- | --- | --- | --- | --- | --- | --- | --- | --- |
| ***Neo02*** | **TP53** | stopgain | 7578212 | G | A | p.R81X | 37 | | 0.63 | 0.81 | 4.75 | 0.84 | 0.46 | 0.03 | 2.95 |
|  | **UTS2R** | nonsynonymous SNV | 80332825 | C | G | p.R209G | 16.3 | | 0.00 | 0.40 | 5.14 | 0.6 | 0.51 | 0.03 | 3.23 |
|  | **ZMYND15** | nonsynonymous SNV | 4647312 | C | T | p.R469W | 10.88 | | 0.88 | 0.79 | 5.9 | 1.1 | 0.48 | 0.09 | 2.31 |
| ***Neo05*** | **ARID1A** | frameshift deletion | 27102082 | TGGCGGG | TGG | p.R1671fs | 0 | | 0.50 | 0.00 | 0 | 0 | NA | NA | 1.89 |
|  | **CIT** | nonsynonymous SNV | 120166366 | G | C | p.R1136G | 17.25 | | 0.10 | 0.26 | 0.88 | 0.43 | NA | NA | 0.11 |
|  | **DHX40** | nonsynonymous SNV | 57643040 | G | A | p.R3Q | 15.76 | | 0.90 | 0.91 | 4 | 1.59 | NA | NA | 0.08 |
|  | **HDAC9** | frameshift deletion | 18788734 | AG | A | p.E670fs | 0 | | 0.70 | 0.00 | 0.68 | 0.05 | NA | NA | 3.17 |
|  | **INSM1** | nonsynonymous SNV | 20349635 | G | T | p.G242W | 17.49 | | 0.97 | 0.85 | 1.74 | 1.56 | NA | NA | 0.38 |
|  | **KIF13B** | nonsynonymous SNV | 28999819 | C | T | p.R730Q | 18.68 | | 0.00 | 0.00 | 0.02 | 0.01 | NA | NA | 0.03 |
|  | **PARK2** | nonsynonymous SNV | 161990410 | A | C | p.F155V | 21.7 | | 0.36 | 1.00 | 0.93 | 0 | NA | NA | 0 |
|  | **ROBO2** | nonsynonymous SNV | 77147279 | C | T | p.T59M | 21.5 | | NA | 0.55 | 0 | 0 | NA | NA | 0 |
|  | **TRPM3** | nonsynonymous SNV | 73168179 | G | T | p.L916M | 16.83 | | 0.00 | 0.99 | 0 | 0 | NA | NA | 0 |
|  | **WIF1** | nonsynonymous SNV | 65445169 | T | G | p.E367A | 13.55 | | 0.13 | 1.00 | 0.36 | 0.12 | NA | NA | 0 |
| ***Neo06*** | **DNAH9** | nonsynonymous SNV | 11778483 | C | T | p.T3487M | 12.4 | | 0.82 | NA | 0.06 | 0.09 | 0.052 | 0.065 | 0.056 |
|  | **HIST1H3C** | nonsynonymous SNV | 26045778 | T | A | p.V47E | 13.37 | | 0.70 | NA | 0.07 | 0.052 | 0.03 | 0.03 | 0.04 |
|  | **RAET1G** | nonsynonymous SNV | 150240839 | C | T | p.G67S | 12.51 | | 0.98 | NA | 0.055 | 0.038 | 0.035 | 0.105 | 0.09 |
|  | **TP53** | nonsynonymous SNV | 7578265 | A | G | p.I63T | 13.73 | | 0.86 | NA | 0.16 | 0.07 | 0.049 | 0.1 | 0.11 |
| ***Neo07*** | **GNA13** | stopgain | 63010731 | G | A | p.R165X | 37 | | 0.88 | 0.97 | NA | 0.27 | 0.2 | 0.085 | 1.08 |
|  | **NFKB1** | nonsynonymous SNV | 103531819 | C | G | p.P771R | 13.44 | | 0.85 | 0.96 | NA | 0.014 | 0 | 0.019 | 1.29 |
|  | **PFKM** | nonsynonymous SNV | 48533646 | A | G | p.N381S | 20.9 | | 0.90 | 0.91 | NA | 0.28 | 0.22 | 0.2 | 1.89 |
|  | **TP53** | nonsynonymous SNV | 7577129 | A | G | p.F138S | 22.2 | | 0.92 | 0.91 | NA | 0.12 | 0.16 | 0.16 | 1.89 |
| ***Neo08*** | **FLCN** | nonsynonymous SNV | 17131419 | G | C | p.C11W | 11.2 | | 0.17 | NA | 0.69 | 0.005 | 0.019 | 0 | 0.038 |
| ***Neo12*** | **COL27A1** | nonsynonymous SNV | 116940623 | G | C | p.R654P | 12.16 | | 0.19 | NA | 0.25 | 0 | 0 | 0 | 0 |
|  | **MROH7** | stopgain | 55172120 | C | T | p.R711X | 41 | | 0.23 | NA | 0.1 | 0.046 | 0.034 | 0.028 | 0.041 |
|  | **MRPL2** | frameshift deletion | 43024094 | ACTTGGTCTCCTC | AC | p.E115fs | 0 | | 0.15 | NA | 0 | 0 | 0 | 0 | 0 |
|  | **SACS** | frameshift deletion | 23927942 | CCTTT | C | p.L574fs | 0 | | 0.14 | NA | 0 | 0 | 0 | 0 | 0 |
| ***Neo14*** | **HTR5A** | nonsynonymous SNV | 154863025 | G | A | p.R139H | 34 | | 0.18 | NA | 1.2 | 0.11 | 0.11 | 0.24 | 0.08 |
|  | **RB1** | frameshift deletion | 48955463 | GATTT | G | p.D527fs | 0 | | 0.15 | NA | 1.43 | 0 | 0 | 0 | 0 |
|  | **TP53** | nonsynonymous SNV | 7577539 | G | A | p.R116W | 18.84 | | 0.20 | NA | 0.85 | 0.08 | 0.015 | 0.016 | 0.019 |
| ***Neo17*** | **HLF** | nonsynonymous SNV | 53398125 | C | G | p.S258W | 13.98 | | NA | 0.51 | 49 | 5 | 0 | 0.29 | 5.3 |
|  | **PKP3** | stopgain | 400619 | G | T | p.E551X | 33 | | NA | 0.67 | 52 | 3.4 | 0 | 0 | 5.7 |
|  | **SALL3** | nonsynonymous SNV | 76753147 | G | A | p.A386T | 16.06 | | NA | 0.95 | 20.8 | 2.26 | 0.9 | 2.6 | 4.8 |
|  | **TNC** | nonsynonymous SNV | 117797608 | C | T | p.D1888N | 25.3 | | NA | 0.44 | 15 | 4.18 | 2.05 | 0.72 | 3.4 |
|  | **TP53** | frameshift deletion | 7578505 | GGGCAGGTCTTGGCCAGTTGGCAAAACATCTTGT | G | p.P142fs | 0 | | NA | 0.97 | 45.7 | 5.4 | 3.8 | 1.7 | 5.1 |
| ***Neo21*** | **ARFRP1** | nonsynonymous SNV | 62338365 | T | G | p.N27H | 31 | | 0.38 | NA | 4.5 | 0.04 | NA | NA | 0 |
|  | **CNTN6** | nonsynonymous SNV | 1394113 | C | G | p.N418K | 8.106 | | 0.45 | NA | 1.81 | 0.037 | NA | NA | 0 |
|  | **TP53** | nonsynonymous SNV | 7577094 | G | A | p.R150W | 20.8 | | 0.40 | NA | 2.5 | 0.12 | NA | NA | 0 |
|  | **TRIM72** | nonsynonymous SNV | 31235541 | C | T | p.P300L | 18.52 | | 0.46 | NA | 3.1 | 0.17 | NA | NA | 0 |
| ***Neo22*** | **DHRS3** | nonsynonymous SNV | 12628398 | A | C | p.C294G | 18.84 | | 0.38 | NA | 0.13 | 0 | 0 | 0 | 0 |
|  | **GCN1L1** | nonsynonymous SNV | 120594789 | G | A | p.P1032L | 23.8 | | 0.43 | NA | 0.008 | 0.1 | 0.09 | 0.036 | 0 |
|  | **PDZRN4** | nonsynonymous SNV | 41946493 | G | C | p.K155N | 20.7 | | 0.38 | NA | 0.06 | 0 | 0 | 0 | 0 |
|  | **RAB21** | nonsynonymous SNV | 72149007 | C | G | p.T33R | 33 | | 0.39 | NA | 0 | 0 | 0.017 | 0 | 0.017 |
|  | **TP53** | nonsynonymous SNV | 7578268 | A | C | p.L62R | 16.11 | | 0.57 | NA | 0.007 | 0 | 0 | 0 | 0.035 |
| ***Neo24*** | **ADCK4** | nonsynonymous SNV | 41206259 | G | A | p.P290S | 16.75 | | 0.31 | 0.50 | 0.36 | 0.47 | 0.11 | 0.031 | 0.031 |
|  | **AOC3** | nonsynonymous SNV | 41003817 | C | T | p.R153W | 16.73 | | 0.43 | 0.42 | 0.41 | 0.58 | 0.19 | 0.034 | 0.06 |
|  | **MTX2** | nonsynonymous SNV | 177188143 | G | T | p.L53F | 18.24 | | 0.43 | 0.47 | 2.09 | 1.67 | 0 | 0.05 | 0.12 |
|  | **TP53** | nonsynonymous SNV | 7578455 | C | G | p.A27P | 16.75 | | 0.30 | 0.40 | 0.64 | 0.57 | 0 | 0 | 0.047 |
| ***Neo25*** | **ALKBH5** | nonsynonymous SNV | 18111631 | G | A | p.R369H | 26.5 | | 0.30 | 0.24 | 0.25 | 0 | 0.06 | 0.1 | 0.09 |
|  | **COL14A1** | nonsynonymous SNV | 121326208 | G | A | p.G1498E | 20.8 | | 0.32 | 0.30 | 0.44 | 0 | 0 | 0 | 0.31 |
|  | **GRM7** | nonsynonymous SNV | 7721947 | C | T | p.A888V | 14.94 | | 0.58 | 0.33 | 0.48 | 0.03 | 0.04 | 0.036 | 0.036 |
|  | **PLXNA3** | nonsynonymous SNV | 153688974 | A | G | p.M151V | 15.59 | | 0.41 | 0.26 | 1.1 | 0 | 0.2 | 0.19 | 0.51 |
|  | **TMPRSS2** | nonsynonymous SNV | 42861469 | C | G | p.G134A | 0.056 | | 0.56 | 0.34 | 0.44 | 0 | 0 | 0 | 0.41 |
|  | **TP53** | stopgain | 7578212 | G | A | p.R81X | 37 | | 0.38 | 0.40 | 0.25 | 0.33 | 0.17 | 0.22 | 0.25 |
| ***Neo26*** | **CNTNAP5** | nonsynonymous SNV | 125671824 | C | A | p.Q1294K | 24.8 | | 0.60 | NA | 0.13 | 0 | 0 | 0 | 0 |
|  | **PDE4C** | nonsynonymous SNV | 18332966 | C | T | p.R105Q | 34 | | 0.43 | NA | 0.34 | 0.1 | 0.08 | 0.022 | 0.05 |
|  | **SMC3** | nonsynonymous SNV | 112356189 | G | C | p.G666A | 17.39 | | 0.41 | NA | 0.27 | 0 | 0 | 0 | 0 |
|  | **TP53** | nonsynonymous SNV | 7577094 | G | A | p.R150W | 20.8 | | 0.52 | NA | 0.39 | 0.039 | 0.11 | 0.04 | 0.02 |
| ***Neo27*** | **NOL7** | nonsynonymous SNV | 13615603 | C | G | p.R5G | 13.13 | | 0.00 | 0.55 | 0 | 0 | 0 | 0.78 | 0.017 |
|  | **PRRG3** | nonsynonymous SNV | 150868561 | G | A | p.R34Q | 27.9 | | 0.27 | 0.57 | 1.11 | 0.06 | 0.013 | 1.82 | 0.43 |
|  | **ROBO3** | nonsynonymous SNV | 124744718 | G | T | p.W662C | 16.08 | | 0.00 | 0.69 | 1.48 | 0.06 | 0 | 1.79 | 0.28 |
|  | **TP53** | nonsynonymous SNV | 7578413 | C | T | p.V41M | 23.4 | | 0.21 | 0.43 | 0.63 | 0.117 | 0 | 0.49 | 0.138 |
| ***Neo28*** | **GON4L** | stopgain | 155730429 | G | A | p.R1639X | 43 | | 0.57 | 0.49 | 0.25 | 0.09 | 0.07 | 0.11 | 0.11 |
|  | **PLEKHH1** | nonsynonymous SNV | 68046546 | G | A | p.D1046N | 16.96 | | 0.57 | 0.89 | 0.12 | 0.12 | 0.09 | 0.045 | 0.07 |
|  | **SMARCA2** | nonsynonymous SNV | 2116002 | C | T | p.R1213W | 16.95 | | 0.55 | 0.64 | 0.23 | 0.11 | 0.05 | 0.016 | 0.072 |
|  | **SPTLC2** | nonsynonymous SNV | 78028804 | T | A | p.N262I | 23.2 | | 0.87 | 0.87 | 0.2 | 0 | 0.03 | 0.07 | 0.05 |
| ***Neo30*** | **HIST1H1B** | nonsynonymous SNV | 27834942 | T | G | p.K122N | 9.898 | | 0.20 | 0.35 | 0.38 | 0 | 0 | 0.029 | 0.017 |
|  | **MAGI2** | nonsynonymous SNV | 79082626 | C | T | p.S4N | 15.5 | | 0.28 | 0.39 | 0.44 | 0.2 | 0.022 | 0.017 | 0.19 |
|  | **N4BP2L2** | nonsynonymous SNV | 33110825 | C | T | p.D114N | 12.27 | | 0.82 | 0.81 | 0.8 | 0.46 | 0.13 | 0.04 | 0.52 |
|  | **PDE8B** | nonsynonymous SNV | 76703224 | G | A | p.R339H | 16.9 | | 0.43 | 0.75 | 0.81 | 0.33 | 0.4 | 0.16 | 0.36 |
|  | **PSMD1** | nonsynonymous SNV | 231951838 | A | T | p.D609V | 24 | | 0.26 | 0.57 | 0.65 | 0.06 | 0.026 | 0.06 | 0.08 |
|  | **RP1** | nonsynonymous SNV | 55540596 | A | T | p.N1385I | 12.49 | | 0.61 | 0.51 | 1.24 | 0.38 | 0.04 | 0.11 | 0.6 |
|  | **TP53** | frameshift deletion | 7574029 | CGG | CG | p.I200fs | 0 | | 0.70 | 0.99 | 0.82 | 0.39 | 0.017 | 0.46 | 0.38 |
| ***Neo35*** | **EVC2** | nonsynonymous SNV | 5627598 | A | T | p.L562M | 11.87 | | 0.34 | NA | 0.043 | 0.018 | 0.018 | 0.017 | 0.066 |
|  | **GALNT9** | nonsynonymous SNV | 132685715 | A | T | p.V86E | 27.2 | | 0.31 | NA | 0.044 | 0.06 | 0.038 | 0.057 | 0.06 |
|  | **DCX** | stopgain | 110653512 | G | A | p.R120X | 34 | | 0.30 | NA | 0.16 | 0.03 | 0.14 | 0.04 | 0.06 |
|  | **TP53** | Frameshift insertion | 7579358 | CGG | CGGTTTGG | p.F70fs | 0 | | 0.37 | NA | 0 | 0 | 0 | 0 | 0 |
| ***NEO38*** | **KIF19** | nonsynonymous SNV | 72342577 | C | A | p.L280M | 19.54 | | NA | 0.95 | 16 | 0.39 | 0.368 | 0.189 | 0.136 |
|  | **MAP7D3** | nonsynonymous SNV | 135310836 | C | T | p.R576H | 16.41 | | NA | 0.36 | 6.5 | 0.35 | 0.167 | 0.125 | 0.15 |
|  | **ROBO2** | nonsynonymous SNV | 77612365 | G | C | p.G523R | 32 | | 0.00 | 0.97 | 12.09 | 0.42 | 0.082 | 0.26 | 0.127 |
| ***Neo39*** | **ACTL7B** | nonsynonymous SNV | 111618005 | G | T | p.P69Q | 11.53 | | 0.00 | 0.41 | 0.09 | 0 | 0 | 0 | 0 |
|  | **DNAH2** | stopgain | 7721017 | C | T | p.R3387X | 51 | | 0.51 | 0.44 | 0.41 | 0.065 | 0.045 | 0.039 | 0.094 |
|  | **DNAH7** | nonsynonymous SNV | 196866537 | T | A | p.K345N | 19 | | 0.00 | 0.63 | 0.04 | 0.02 | 0 | 0 | 0.01 |
|  | **GRIA1** | nonsynonymous SNV | 153149938 | G | A | p.G665S | 29.5 | | 0.89 | 1.02 | 1.03 | 0.045 | 0.031 | 0.059 | 0.035 |
|  | **GRIN3A** | nonsynonymous SNV | 104499853 | G | A | p.R137C | 21.2 | | 0.40 | 0.42 | 0.06 | 0.05 | 0.04 | 0.1 | 0.05 |
|  | **MAP3K4** | splicing | 161530948 | T | C | NA | 18.5 | | 0.38 | 0.82 | 0.12 | 0.15 | 0.1 | 0.1 | 0.3 |
|  | **RASA1** | nonsynonymous SNV | 86633905 | G | C | p.E338D | 13.61 | | 0.72 | 0.00 | 0 | 0 | 0 | 0 | 0 |
|  | **ROBO1** | nonsynonymous SNV | 78656056 | C | A | p.R1424I | 16.42 | | 0.37 | 0.28 | 0 | 0 | 0 | 0 | 0 |
|  | **SBF2** | nonsynonymous SNV | 9850968 | G | T | p.S1243Y | 17.2 | | 0.00 | 0.54 | 0 | 0 | 0 | 0 | 0 |
|  | **SLC25A4** | nonsynonymous SNV | 186066111 | G | A | p.G102D | 18.23 | | 0.41 | 0.00 | 0.02 | 0.015 | 0.018 | 0.011 | 0 |
|  | **TP53** | nonsynonymous SNV | 7577085 | C | T | p.E153K | 26.1 | | 1.01 | 1.00 | 0.56 | 0.032 | 0.032 | 0.025 | 0.008 |
|  | **UNC5C** | nonsynonymous SNV | 96256773 | A | G | p.F45S | 14.42 | | 0.60 | 0.68 | 0.06 | 0.22 | 0.2 | 0.22 | 0.21 |
| ***Neo40*** | **AGBL1** | stopgain | 86813199 | C | T | p.Q584X | 35 | | 0.91 | NA | 2.9 | NA | NA | NA | 0 |
|  | **PRDM15** | nonsynonymous SNV | 43279786 | C | A | p.D53Y | 25 | | 0.57 | NA | 3.7 | NA | NA | NA | 0 |
|  | **RB1** | stopgain | 48947596 | C | T | p.Q395X | 37 | | 0.68 | NA | 2.79 | NA | NA | NA | 0 |
|  | **TP53** | nonsynonymous SNV | 7577538 | C | T | p.R116P | 27 | | 1.06 | NA | 5.4 | NA | NA | NA | 0.1 |
| ***Neo42*** | **FMN1** | nonsynonymous SNV | 33359085 | T | A | p.D334V | 11.69 | | 0.41 | 0.02 | 0.13 | 0.06 | NA | NA | 0 |
|  | **GNAT1** | nonsynonymous SNV | 50231235 | G | A | p.E167K | 36 | | 0.26 | 0.65 | 0.05 | 0.03 | NA | NA | 0.04 |
|  | **SLC16A2** | nonsynonymous SNV | 73751248 | C | T | p.L494F | 18.9 | | 0.42 | 0.44 | 0.08 | 0.021 | NA | NA | 0 |
|  | **TP53** | nonsynonymous SNV | 7577538 | C | T | p.R116Q | 27.3 | | 0.09 | 0.41 | 0.103 | 0.005 | NA | NA | 0 |
|  | **ZFP36L1** | nonsynonymous SNV | 69256851 | T | C | p.H139R | 20.8 | | 0.28 | 0.72 | 0.19 | 0.1 | NA | NA | 0.35 |
| ***Neo44*** | **ATR** | frameshift deletion | 142259749 | CAACACAATTCAGGAAA | CAA | p.F1188fs | 0 | | 0.33 | NA | 1.57 | 0 | 0 | 0 | 0.013 |
|  | **CASP8** | nonsynonymous SNV | 202149989 | G | T | p.G403V | 20.6 | | 0.58 | NA | 4.22 | 0 | 0 | 0 | 0.014 |
|  | **FGFR2** | nonsynonymous SNV | 123239506 | T | G | p.E665D | 11.73 | | 0.55 | NA | 3.92 | 0.003 | 0 | 0.002 | 0 |
|  | **HPN** | nonsynonymous SNV | 35556775 | G | A | p.D352N | 25.5 | | 0.81 | NA | 7 | 0.11 | 0.07 | 0.048 | 0.033 |
|  | **TP53** | nonsynonymous SNV | 7579355 | A | T | p.L72Q | 21.3 | | 0.63 | NA | 16 | 0.039 | 0.08 | 0.041 | 0.054 |
| ***Neo49*** | **HIST1H2BF** | nonsynonymous SNV | 26199897 | C | G | p.S37R | 11.8 | | 0.42 | NA | 0 | NA | 0 | 0 | 0 |
|  | **HIST1H2BJ** | nonsynonymous SNV | 27100188 | C | G | p.E114D | 12.48 | | 0.33 | NA | 0 | NA | 0 | 0 | 0 |
|  | **TADA2A** | nonsynonymous SNV | 35800727 | G | A | p.E136K | 21.7 | | 0.80 | NA | 0.46 | NA | 0.028 | 0.007 | 0.023 |
|  | **TNFRSF18** | nonsynonymous SNV | 1139821 | G | T | p.T119N | 14.83 | | 0.75 | NA | 0 | NA | 0 | 0.019 | 0.15 |
| ***Neo50*** | **EXOC4** | nonsynonymous SNV | 133692517 | G | T | p.R872S | 20.2 | | 0.42 | 0.43 | 0.78 | 0 | 0.01 | 0.02 | 0.048 |
|  | **GPR158** | nonsynonymous SNV | 25887599 | C | T | p.T1015I | 9.838 | | 0.49 | 0.50 | 0.98 | 0.13 | 0.035 | 0 | 0 |
|  | **RNF123** | nonsynonymous SNV | 49758242 | A | G | p.Y1218C | 17.31 | | 0.42 | 0.52 | 0.4 | 0.13 | 0.1 | 0.039 | 0.062 |
|  | **TP53** | stopgain | 7579528 | C | T | p.W14X | 14.68 | | 0.63 | 0.63 | 0.41 | 0.023 | 0.042 | 0.011 | 0.17 |
| ***Neo57*** | **ANKRD12** | nonsynonymous SNV | 9275559 | G | T | p.R1911L | 35 | | 0.78 | NA | 1.35 | 1.69 | 0.042 | 0 | 0 |
|  | **BAP1** | nonsynonymous SNV | 52441263 | G | C | p.H169Q | 18.26 | | 0.90 | NA | 1.86 | 3.35 | 0 | 0 | 0 |
|  | **NR5A1** | nonsynonymous SNV | 127255388 | T | A | p.E304V | 25.4 | | 0.89 | NA | 1.63 | 2.05 | 0.051 | 0.041 | 0.032 |
|  | **TFCP2** | nonsynonymous SNV | 51566135 | G | T | p.A24D | 28.6 | | 0.84 | NA | 1.23 | 1.98 | 0 | 0 | 0 |
| ***Neo58*** | **DNAJC12** | nonsynonymous SNV | 69571328 | G | A | p.S84L | 14.62 | | 0.48 | NA | 0.06 | 0.075 | 0.078 | 0.067 | 0.073 |
|  | **HIPK2** | frameshift deletion | 139311410 | ATTGGAGTGATTCTCTTG | ATTG | p.D513fs | 0 | | 0.36 | NA | 0 | 0 | 0 | 0 | 0 |
|  | **YEATS2** | nonsynonymous SNV | 183476666 | G | T | p.K523N | 16.87 | | 0.71 | NA | 0 | 0 | 0 | 0 | 0 |
|  | **ZDHHC2** | nonsynonymous SNV | 17067483 | A | G | p.N258S | 16.51 | | 0.45 | NA | 0.115 | 0.098 | 0.128 | 0.116 | 0.108 |
|  |  | Non-detectable values (under ToD) | | | | |  | |  |  |  |  |  |  |  |

**Supplementary Table S5- MAF of all tested variants in pre- and post-NAC tumor and T0-T4 plasma.**

| ***Patients*** | ***Gene*** | ***RCB score*** | ***Date of surgery*** | ***Date of 1st post-op blood*** | ***Interval (Months)*** | ***T4 MAF*** | ***1st post op MAF*** | |
| --- | --- | --- | --- | --- | --- | --- | --- | --- |
| ***Neo02*** | **TP53** | RCB3 | 09-May-11 | 28-Jul-11 | **2.6** | 2.95 | 1.45 | |
|  | **UTS2R** | RCB3 | 09-May-11 | 28-Jul-11 | **2.6** | 3.23 | 0.88 | |
|  | **ZMYND15** | RCB3 | 09-May-11 | 28-Jul-11 | **2.6** | 2.31 | 2.21 | |
| ***Neo08*** | **FLCN** | RCB0 | 22-Mar-12 | 24-Apr-12 | **1** | 0.038 | ND | |
| ***Neo12*** | **MROH7** | RCB0 | 13-Sep-12 | 17-Oct-12 | **1.1** | ND | ND | |
| ***Neo17*** | **TNC** | RCB3 | 06-Aug-12 | 04-Sep-12 | **1** | 3.4 | 38.9 | |
|  | **SALL3** | RCB3 | 06-Aug-12 | 04-Sep-12 | **1** | 4.8 | 59 | |
|  | **TP53** | RCB3 | 06-Aug-12 | 04-Sep-12 | **1** | 5.1 | 75.1 | |
|  | **HLF** | RCB3 | 06-Aug-12 | 04-Sep-12 | **1** | 5.3 | 69.2 | |
|  | **PKP3** | RCB3 | 06-Aug-12 | 04-Sep-12 | **1** | 5.7 | 44.9 | |
| ***Neo25*** | **ALKBH5** | RCB3 | 17-Jan-13 | 12-Mar-13 | **1.8** | 0.09 | 2.7 | |
|  | **COL14A1** | RCB3 | 17-Jan-13 | 12-Mar-13 | **1.8** | 0.31 | 1.36 | |
|  | **TMPRSS2** | RCB3 | 17-Jan-13 | 12-Mar-13 | **1.8** | 0.41 | 4.99 | |
|  | **GRM7** | RCB3 | 17-Jan-13 | 12-Mar-13 | **1.8** | ND | 6.4 | |
|  | **TP53** | RCB3 | 17-Jan-13 | 12-Mar-13 | **1.8** | 0.25 | 5.45 | |
|  | **PLXNA3** | RCB3 | 17-Jan-13 | 12-Mar-13 | **1.8** | 0.51 | 5.6 | |
| ***Neo38*** | **MAP7D3** | RCB3 | 23-Apr-13 | 29-May-13 | **1.2** | ND | 0.71 | |
|  | **ROBO2** | RCB3 | 23-Apr-13 | 29-May-13 | **1.2** | 0.127 | 0.52 | |
|  | **KIF19** | RCB3 | 23-Apr-13 | 29-May-13 | **1.2** | ND | 0.88 | |
| ***Neo42*** | **TP53** | RCB3 | 30-May-13 | 25-Jun-13 | **0.9** | ND | ND | |
|  | **FMN1** | RCB3 | 30-May-13 | 25-Jun-13 | **0.9** | ND | 0.028 | |
| ***Neo50*** | **RNF123** | RCB2 | 20-Jun-13 | 31-Jul-13 | **1.3** | ND | ND | |
|  | **EXOC4** | RCB2 | 20-Jun-13 | 31-Jul-13 | **1.3** | 0.048 | ND | |
|  | **GPR158** | RCB2 | 20-Jun-13 | 31-Jul-13 | **1.3** | ND | ND | |
| ***Neo57*** | **TFCP2** | RCB1 | 27-Feb-14 | 26-May-14 | **2.9** | ND | ND | |
|  | **ANKRD12** | RCB1 | 27-Feb-14 | 26-May-14 | **2.9** | ND | ND | |
|  | **NR5A1** | RCB1 | 27-Feb-14 | 26-May-14 | **2.9** | ND | ND | |
|  | **BAP1** | RCB1 | 27-Feb-14 | 26-May-14 | **2.9** | ND | ND | |
| ***Neo58*** | **DNAJC12** | RCB0 | 06-Mar-14 | 07-Apr-14 | **1.1** | ND | ND | |
|  | **ZDHHC2** | RCB0 | 06-Mar-14 | 07-Apr-14 | **1.1** | ND | ND | |
| ***Average*** | | | | | | 2.16 | | 17.81 |
| ***Standard Deviation*** | | | | | | 2.18 | | 26.43 |
| ***Median*** | | | | | | 1.41 | | 3.85 |
| ***Min-Max*** | | | | | | 0.04-5.70 | | 0.28-75.10 |

**Supplementary Table S6- Comparison of pre and post-operative ctDNA MAF values.**

| ***Sample*** | ***Gene*** | ***Variation*** | ***Position*** | ***Ref*** | ***Alt*** | ***Protein Change*** | ***CADDa*** | ***Pre-NAC***  ***Tumor MAF*** | ***Post-NAC***  ***Tumor MAF*** | ***T0*** | ***T1*** | ***T2*** | ***T3*** | ***T4*** |
| --- | --- | --- | --- | --- | --- | --- | --- | --- | --- | --- | --- | --- | --- | --- |
| ***Neo02*** | **TP53** | stopgain | 7578212 | G | A | p.R81X | 37 | 0.63 | 0.81 | 4.75 | 0.84 | 0.46 | 0.03 | 2.95 |
| ***Neo06*** | **TP53** | nonsynonymous SNV | 7578265 | A | G | p.I63T | 13.73 | 0.86 | NA | 0.16 | 0.07 | 0.049 | 0.1 | 0.11 |
| ***Neo07*** | **TP53** | nonsynonymous SNV | 7577129 | A | G | p.F138S | 22.2 | 0.92 | 0.91 | NA | 0.12 | 0.16 | 0.16 | 1.89 |
| ***Neo14*** | **TP53** | nonsynonymous SNV | 7577539 | G | A | p.R116W | 18.84 | 0.20 | NA | 0.85 | 0.08 | 0.015 | 0.016 | 0.019 |
| ***Neo17*** | **TP53** | frameshift deletion | 7578505 | GGGCAGGTCTTGGCCAGTTGGCAAAACATCTTGT | G | p.P142fs | 0 | NA | 0.97 | 45.7 | 5.4 | 3.8 | 1.7 | 5.1 |
| ***Neo21*** | **TP53** | nonsynonymous SNV | 7577094 | G | A | p.R150W | 20.8 | 0.40 | NA | 2.5 | 0.12 | NA | NA | 0 |
| ***Neo22*** | **TP53** | nonsynonymous SNV | 7578268 | A | C | p.L62R | 16.11 | 0.57 | NA | 0.007 | 0 | 0 | 0 | 0.035 |
| ***Neo24*** | **TP53** | nonsynonymous SNV | 7578455 | C | G | p.A27P | 16.75 | 0.30 | 0.40 | 0.64 | 0.57 | 0 | 0 | 0.047 |
| ***Neo25*** | **TP53** | stopgain | 7578212 | G | A | p.R81X | 37 | 0.38 | 0.40 | 0.25 | 0.33 | 0.17 | 0.22 | 0.25 |
| ***Neo26*** | **TP53** | nonsynonymous SNV | 7577094 | G | A | p.R150W | 20.8 | 0.52 | NA | 0.39 | 0.039 | 0.11 | 0.04 | 0.02 |
| ***Neo27*** | **TP53** | nonsynonymous SNV | 7578413 | C | T | p.V41M | 23.4 | 0.21 | 0.43 | 0.63 | 0.117 | 0 | 0.49 | 0.138 |
| ***Neo30*** | **TP53** | frameshift deletion | 7574029 | CGG | CG | p.I200fs | 0 | 0.70 | 0.99 | 0.82 | 0.39 | 0.017 | 0.46 | 0.38 |
| ***Neo35*** | **TP53** | frameshift insertion | 7579358 | CGG | CGGTTTGG | p.F70fs | 0 | 0.37 | NA | 0 | 0 | 0 | 0 | 0 |
| ***Neo39*** | **TP53** | nonsynonymous SNV | 7577085 | C | T | p.E153K | 26.1 | 1.01 | 1.00 | 0.56 | 0.032 | 0.032 | 0.025 | 0.008 |
| ***Neo40*** | **TP53** | nonsynonymous SNV | 7577538 | C | T | p.R116P | 27 | 1.06 | NA | 5.4 | NA | NA | NA | 0.1 |
| ***Neo42*** | **TP53** | nonsynonymous SNV | 7577538 | C | T | p.R116Q | 27.3 | 0.09 | 0.41 | 0.103 | 0.005 | NA | NA | 0 |
| ***Neo44*** | **TP53** | nonsynonymous SNV | 7579355 | A | T | p.L72Q | 21.3 | 0.63 | NA | 16 | 0.039 | 0.08 | 0.041 | 0.054 |
| ***Neo50*** | **TP53** | stopgain | 7579528 | C | T | p.W14X | 14.68 | 0.63 | 0.63 | 0.41 | 0.023 | 0.042 | 0.011 | 0.17 |
|  |  | Non-detectable values (under ToD) | | | | |  |  |  |  |  |  |  |  |

**Supplementary Table S7- MAF of TP53 variants in pre- and post-NAC tumor and T0-T4 plasma.**

| **Clinical characteristics** | | | **Patients, n (%)** | **Average MAF at baseline for TP53 variants (%)** | **p- value** |
| --- | --- | --- | --- | --- | --- |
| **Patients** | Age, years | ≤50  >50 | 9 (50) | 2.85 | 0.5311 |
|  |  |  | 9 (50) | 6.69 |  |
|  | BRCA 1/2 mutated* | Positive  Negative | 2 (50) | 5.4 | Only 1 value |
|  |  |  | 2 (50) | 2.12 |  |
| **Pre-NAC** (Neoadjuvant chemotherapy) **tumor** | cT | 1-2  3-4 | 14 (78) | 2.31 | 0.4377 |
|  |  |  | 4 (22) | 12.29 |  |
|  | cN | 0  1-3 | 10 (56) | 2.60 | 0.4744 |
|  |  |  | 8 (44) | 6.97 |  |
|  | Stage | I  II  III | 1 (6) | 0 | No value |
|  |  |  | 13 (72) | 2.50 | 0.4459 |
|  |  |  | 4 (22) | 12.29 |  |
|  | Grade | 1  2  3 | 0 (0) | 0 | No value |
|  |  |  | 4 (22) | 0.38 | 0.1389 |
|  |  |  | 14 (78) | 5.57 |  |
|  | Histological type** | Ductal  Lobular | 17 (85) | 4.94 | 0.5632 |
|  |  |  | 3 (15) | 15.40 |  |
| **Treatment** | NAC drugs regimen | Only taxane  AT  TA  Other | 3 (17) | 0.49 | 0.1470 |
|  |  |  | 13 (72) | 5.96 |  |
|  |  |  | 1 (6) | 0.64 | Only 1 value |
|  |  |  | 1 (6) | 0.10 |  |
| **Post-NAC tumor***** | pN | Negative  Positive | 11 (65) | 2.65 | 0.4643 |
|  |  |  | 6 (35) | 8.64 |  |
|  | Residual tumor size (cm) | 0  ≤2  >2 | 5 (29) | 1.36 | 0.6048 |
|  |  |  | 2 (12) | 1.57 |  |
|  |  |  | 10 (59) | 7.60 |  |
|  | RCB | 0  ≥ 1 | 4 (22) | 1.60 | 0.3076 |
|  |  |  | 14 (78) | 5.60 |  |
|  |  | 0-1  2-3 | 6 (33) | 1.55 | 0.2739 |
|  |  |  | 12 (67) | 6.46 |  |

**Supplementary Table S8- Association of Average Mutant Allele Frequencies in TP53 gene variants at baseline (T0) with patient and tumor characteristics.** Average MAF of TP53 gene variants according to different patient characteristics (age, BRCA1/2 status), pre-neoadjuvant chemotherapy (pre-NAC) tumor characteristics (cT, cN, stage, grade, and histology), treatment (NAC), or post-NAC tumor characteristics (pN, residual tumor size, RCB score). Stage (cT, cN and stage) is according to the AJCC 8th edition. NAC drug regimens were classified as only taxanes, anthracycline before (AT) or after (TA) taxanes, or other regimens. Average MAF is compared with a two-tailed Student’s t test when there were 2 independent groups and with an ANOVA (Analysis of variance) test when there were more than 2. Grey shading is added when there are not enough values to do the calculation and there are no p values <0.05. *BRCA1/2 status: Unknown clinical data for 14, so we have n=4. ** 2 patients had lobular and ductal breast cancer, so we have n=20. *** One patient had no surgery due to the development of metastatic disease, so we have n =17 at the surgery for pN and residual tumor size. This patient was assigned a RCB score =3. cT: clinical tumor size; cN: clinical nodal satus; pN: pathological nodal status.


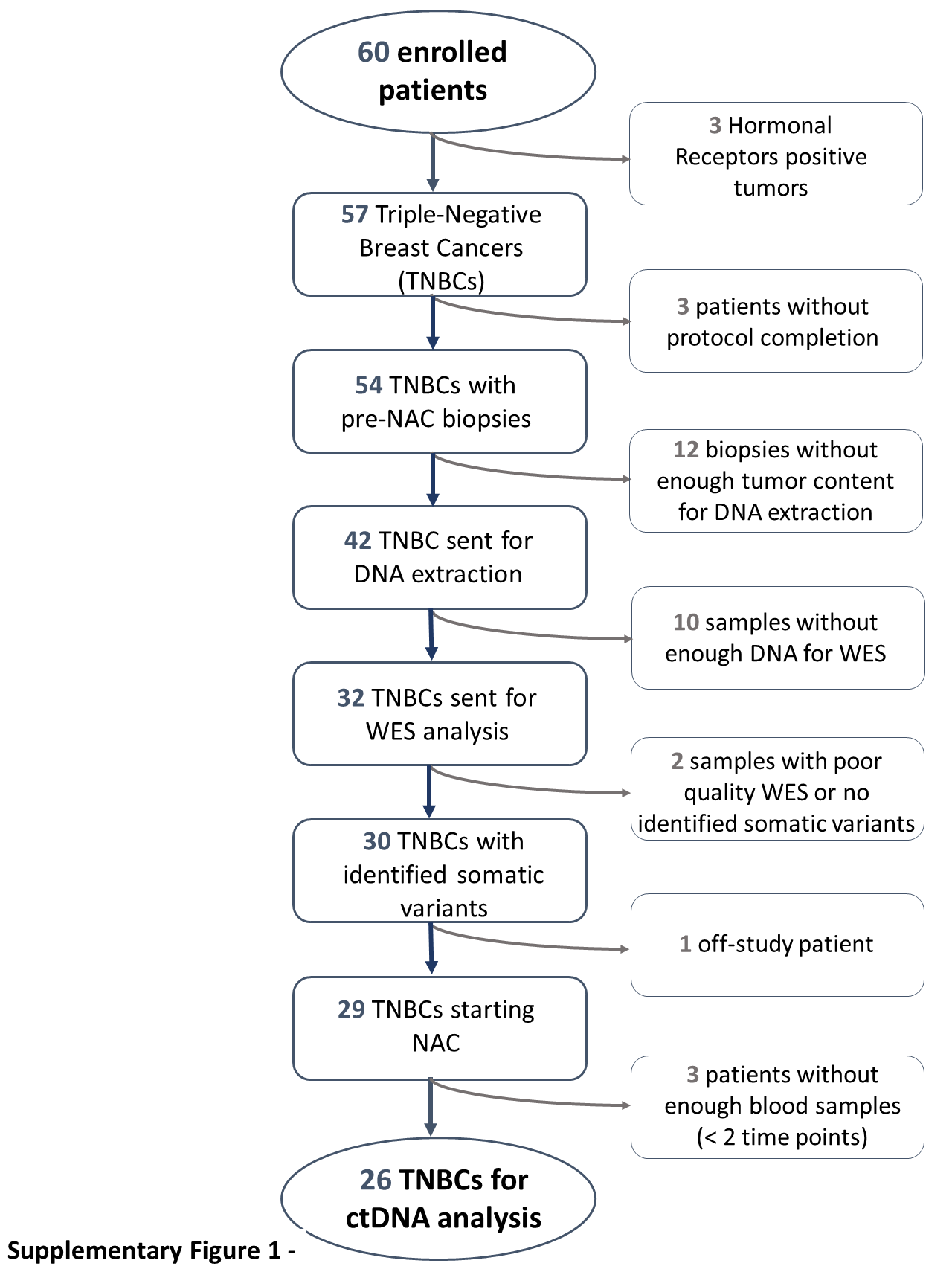


**Supplementary Figure S1-Flow Chart of Q-CROC-03 patient inclusion in ctDNA testing cohort.** Of 60 total patients enrolled in Q-CROC-03, 3 were later found to be positive for either Estrogen Receptor (ER>10%), or Progesterone Receptor (PR>10%), 3 did not complete the protocol and thus did not get their biopsies analyzed, 12 had biopsies without enough tumor content for DNA extraction (too much necrosis or not enough cellularity), 10 did not have enough extracted DNA to perform Whole Exome Sequencing (WES), and 2 had either poor quality WES or no reliably identified somatic variants from WES. One patient was excluded because of comorbidities, and 3 patients were then excluded because they had only one blood collection time point available for analysis.


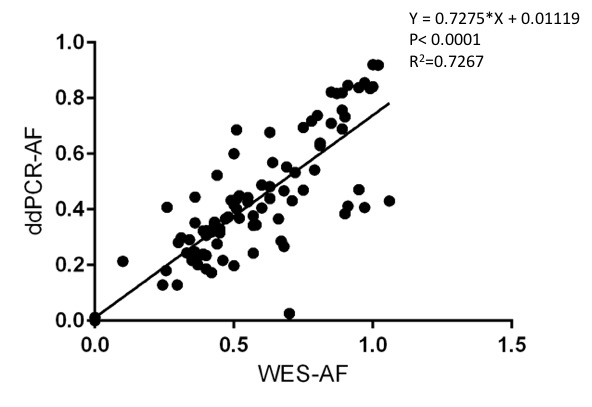


**Supplementary Figure S2- Correlation between ddPCR and WES variant allele frequencies in tumors.**

Degree of Pearson correlation between the mutant allele frequency (MAF) of SNVs in patient matched tumor DNA measured by ddPCR and WES. Line indicates y = x.

**Supplementary Figure S3- Tumor MAF in pCR and non-pCR patients.**
